# 2017 HRS/EHRA/ECAS/APHRS/SOLAECE expert consensus statement on catheter and surgical ablation of atrial fibrillation: executive summary

**DOI:** 10.1007/s10840-017-0277-z

**Published:** 2017-09-15

**Authors:** Hugh Calkins, Gerhard Hindricks, Riccardo Cappato, Young-Hoon Kim, Eduardo B. Saad, Luis Aguinaga, Joseph G. Akar, Vinay Badhwar, Josep Brugada, John Camm, Peng-Sheng Chen, Shih-Ann Chen, Mina K. Chung, Jens Cosedis Nielsen, Anne B. Curtis, D. Wyn Davies, John D. Day, André d’Avila, N.M.S. (Natasja) de Groot, Luigi Di Biase, Mattias Duytschaever, James R. Edgerton, Kenneth A. Ellenbogen, Patrick T. Ellinor, Sabine Ernst, Guilherme Fenelon, Edward P. Gerstenfeld, David E. Haines, Michel Haissaguerre, Robert H. Helm, Elaine Hylek, Warren M. Jackman, Jose Jalife, Jonathan M. Kalman, Josef Kautzner, Hans Kottkamp, Karl Heinz Kuck, Koichiro Kumagai, Richard Lee, Thorsten Lewalter, Bruce D. Lindsay, Laurent Macle, Moussa Mansour, Francis E. Marchlinski, Gregory F. Michaud, Hiroshi Nakagawa, Andrea Natale, Stanley Nattel, Ken Okumura, Douglas Packer, Evgeny Pokushalov, Matthew R. Reynolds, Prashanthan Sanders, Mauricio Scanavacca, Richard Schilling, Claudio Tondo, Hsuan-Ming Tsao, Atul Verma, David J. Wilber, Teiichi Yamane

**Affiliations:** 1 Johns Hopkins Medical Institutions, Baltimore, MD USA; 2 Heart Center Leipzig, Leipzig, Germany; 3 Humanitas Research Hospital, Arrhythmias and Electrophysiology Research Center, Milan, Italy; 4 Present Address: Department of Biomedical Sciences, Humanitas University, Milan, Italy; 5 IRCCS, Humanitas Clinical and Research Center, Milan, Italy; 6 Korea University, Seoul, South Korea; 7 Hospital Pro-Cardiaco and Hospital Samaritano, Botafogo, Rio de Janeiro, Brazil; 8 Centro Privado de Cardiología, Tucuman, Argentina; 9 Yale University School of Medicine, New Haven, CT USA; 10 West Virginia University School of Medicine, Morgantown, WV USA; 11 Cardiovascular Institute, Hospital Clínic, University of Barcelona, Catalonia, Spain; 12 St. George’s University of London, London, UK; 13 Indiana University School of Medicine, Indianapolis, IN USA; 14 National Yang-Ming University, Taipei, Taiwan; 15 Cleveland Clinic, Cleveland, OH USA; 16 Aarhus University Hospital, Skejby, Denmark; 17 University at Buffalo, Buffalo, NY USA; 18 Imperial College Healthcare NHS Trust, London, UK; 19 Intermountain Medical Center Heart Institute, Salt Lake City, UT USA; 20 Hospital SOS Cardio, Florianopolis, SC Brazil; 21 Erasmus Medical Center, Rotterdam, The Netherlands; 22 Albert Einstein College of Medicine, Montefiore-Einstein Center for Heart & Vascular Care, Bronx, NY USA; 23 Universitair Ziekenhuis Gent (Ghent University Hospital), Ghent, Belgium; 24 The Heart Hospital, Baylor Plano, Plano, TX USA; 25 Virginia Commonwealth University School of Medicine, Richmond, VA USA; 26 Massachusetts General Hospital, Boston, MA USA; 27 Royal Brompton and Harefield NHS Foundation Trust, National Heart and Lung Institute, Imperial College London, London, UK; 28 Albert Einstein Jewish Hospital, Federal University of São Paulo, São Paulo, Brazil; 29 University of California, San Francisco, San Francisco, CA USA; 30 Beaumont Health System, Royal Oak, MI USA; 31 Hôpital Cardiologique du Haut-Lévêque, Pessac, France; 32 Boston University Medical Center, Boston, MA USA; 33 Boston University School of Medicine, Boston, MA USA; 34 Heart Rhythm Institute, University of Oklahoma Health Sciences Center, Oklahoma City, OK USA; 35 University of Michigan, Ann Arbor, MI USA; 36 The National Center for Cardiovascular Research Carlos III (CNIC), Madrid, Spain; 37 CIBERCV, Madrid, Spain; 38 Royal Melbourne Hospital and University of Melbourne, Melbourne, Australia; 39 Institute for Clinical and Experimental Medicine, Prague, Czech Republic; 40 Hirslanden Hospital, Department of Electrophysiology, Zurich, Switzerland; 41 Asklepios Klinik St. Georg, Hamburg, Germany; 42 Heart RhythmCenter, Fukuoka Sanno Hospital, Fukuoka, Japan; 43 Saint Louis University Medical School, St. Louis, MO USA; 44 Department of Cardiology and Intensive Care, Hospital Munich-Thalkirchen, Munich, Germany; 45 Montreal Heart Institute, Department of Medicine, Université de Montréal, Montréal, Canada; 46 Hospital of the University of Pennsylvania, Philadelphia, PA USA; 47 University of Pennsylvania School of Medicine, Philadelphia, PA USA; 48 Brigham and Women’s Hospital, Boston, MA USA; 49 St. David’s Medical Center, Texas Cardiac Arrhythmia Institute, Austin, TX USA; 50 Montreal Heart Institute, Montreal, QC Canada; 51 Université de Montréal, Montreal, QC Canada; 52 McGill University, Montreal, QC Canada; 53 University Duisburg-Essen, Essen, Germany; 54 Division of Cardiology, Saiseikai Kumamoto Hospital, Kumamoto, Japan; 55 Mayo Clinic, Rochester, MN USA; 56 State Research Institute of Circulation Pathology, Novosibirsk, Russia; 57 Lahey Hospital and Medical Center, Burlington, MA USA; 58 Centre for Heart Rhythm Disorders, South Australian Health and Medical Research Institute, University of Adelaide, Adelaide, Australia; 59 Royal Adelaide Hospital, Adelaide, Australia; 60 Instituto do Coração (InCor), São Paulo, Brazil; 61 Barts Heart Centre, London, UK; 62 Cardiac Arrhythmia Research Center, Centro Cardiologico Monzino, IRCCS, Milan, Italy; 63 Department of Cardiovascular Sciences, University of Milan, Milan, Italy; 64 National Yang-Ming University Hospital, Yilan City, Taiwan; 65 Southlake Regional Health Centre, University of Toronto, Toronto, ON Canada; 66 Loyola University of Chicago, Chicago, IL USA; 67 Jikei University School of Medicine, Tokyo, Japan

**Keywords:** Ablation, Arrhythmia, Atrial fibrillation, Atrial flutter, Atrial tachycardia, Catheter ablation, Surgical ablation, Stroke, Anticoagulation

**Chair**: Hugh Calkins, MD, Johns Hopkins Medical Institutions, Baltimore, MD, USA.

**Section Chairs: Definitions, Mechanisms, and Rationale for AF Ablation**: Shih-Ann Chen, MD, National Yang-Ming University, Taipei, Taiwan.

**Modifiable Risk Factors for AF and Impact on Ablation**: Jonathan M. Kalman, MBBS, PhD, Royal Melbourne Hospital and University of Melbourne, Melbourne, Australia.

**Indications**: Claudio Tondo, MD, PhD, Cardiac Arrhythmia Research Center, Centro Cardiologico Monzino, IRCCS, Department of Cardiovascular Sciences, University of Milan, Milan, Italy.

**Strategies, Techniques, and Endpoints**: Karl Heinz Kuck, MD, PhD, Asklepios Klinik St. Georg, Hamburg, Germany.

**Technology and Tools**: Andrea Natale, MD, Texas Cardiac Arrhythmia Institute, St. David’s Medical Center, Austin, TX, USA.

**Technical Aspects of Ablation to Maximize Safety and Anticoagulation**: David E. Haines, MD, Beaumont Health System, Royal Oak, MI, USA.

**Follow-up Considerations**: Francis E. Marchlinski, MD, Hospital of the University of Pennsylvania, University of Pennsylvania School of Medicine, Philadelphia, PA, USA.

**Outcomes and Efficacy**: Matthew R. Reynolds, MD, MSc, Lahey Hospital and Medical Center, Burlington, MA, USA.

**Complications**: D. Wyn Davies, MD, Imperial College Healthcare NHS Trust, London, United Kingdom.

**Training Requirements**: Bruce D. Lindsay, MD, Cleveland Clinic, Cleveland, OH, USA.

**Surgical and Hybrid AF Ablation**: James R. Edgerton, MD, The Heart Hospital, Baylor Plano, Plano, TX, USA.

**Clinical Trial Design**: Atul Verma, MD, Southlake Regional Health Centre, University of Toronto, Toronto, Canada.

**Correspondence**: Heart Rhythm Society, 1325 G Street NW, Suite 400, Washington, DC 20005. E-mail address: clinicaldocs@hrsonline.org.

**Document Reviewers**: Carina Blomström-Lundqvist, MD, PhD; Angelo A.V. De Paola, MD, PhD; Peter M. Kistler, MBBS, PhD; Gregory Y.H. Lip, MD; Nicholas S. Peters, MD; Cristiano F. Pisani, MD; Antonio Raviele, MD; Eduardo B. Saad, MD, PhD; Kazuhiro Satomi, MD, PhD; Martin K. Stiles, MB ChB, PhD; Stephan Willems, MD, PhD

## Introduction

During the past three decades, catheter and surgical ablation of atrial fibrillation (AF) have evolved from investigational procedures to their current role as effective treatment options for patients with AF. Surgical ablation of AF, using either standard, minimally invasive, or hybrid techniques, is available in most major hospitals throughout the world. Catheter ablation of AF is even more widely available, and is now the most commonly performed catheter ablation procedure.

In 2007, an initial Consensus Statement on Catheter and Surgical AF Ablation was developed as a joint effort of the Heart Rhythm Society (HRS), the European Heart Rhythm Association (EHRA), and the European Cardiac Arrhythmia Society (ECAS) [1]. The 2007 document was also developed in collaboration with the Society of Thoracic Surgeons (STS) and the American College of Cardiology (ACC). This Consensus Statement on Catheter and Surgical AF Ablation was rewritten in 2012 to reflect the many advances in AF ablation that had occurred in the interim [2]. The rate of advancement in the tools, techniques, and outcomes of AF ablation continue to increase as enormous research efforts are focused on the mechanisms, outcomes, and treatment of AF. For this reason, the HRS initiated an effort to rewrite and update this Consensus Statement. Reflecting both the worldwide importance of AF, as well as the worldwide performance of AF ablation, this document is the result of a joint partnership between the HRS, EHRA, ECAS, the Asia Pacific Heart Rhythm Society (APHRS), and the Latin American Society of Cardiac Stimulation and Electrophysiology (Sociedad Latinoamericana de Estimulación Cardíaca y Electrofisiología [SOLAECE]). The purpose of this 2017 Consensus Statement is to provide a state-of-the-art review of the field of catheter and surgical ablation of AF and to report the findings of a writing group, convened by these five international societies. The writing group is charged with defining the indications, techniques, and outcomes of AF ablation procedures. Included within this document are recommendations pertinent to the design of clinical trials in the field of AF ablation and the reporting of outcomes, including definitions relevant to this topic.

The writing group is composed of 60 experts representing 11 organizations: HRS, EHRA, ECAS, APHRS, SOLAECE, STS, ACC, American Heart Association (AHA), Canadian Heart Rhythm Society (CHRS), Japanese Heart Rhythm Society (JHRS), and Brazilian Society of Cardiac Arrhythmias (Sociedade Brasileira de Arritmias Cardíacas [SOBRAC]). All the members of the writing group, as well as peer reviewers of the document, have provided disclosure statements for all relationships that might be perceived as real or potential conflicts of interest. All author and peer reviewer disclosure information is provided in Appendix A Table 14 and Appendix B Table 15.

In writing a consensus document, it is recognized that *consensus* does not mean that there was complete agreement among all the writing group members. Surveys of the entire writing group were used to identify areas of consensus concerning performance of AF ablation procedures and to develop recommendations concerning the indications for catheter and surgical AF ablation. These recommendations were systematically balloted by the 60 writing group members and were approved by a minimum of 80% of these members. The recommendations were also subject to a 1-month public comment period. Each partnering and collaborating organization then officially reviewed, commented on, edited, and endorsed the final document and recommendations.

The grading system for indication of class of evidence level was adapted based on that used by the ACC and the AHA [3, 4]. It is important to state, however, that this document is not a guideline. The indications for catheter and surgical ablation of AF, as well as recommendations for procedure performance, are presented with a Class and Level of Evidence (LOE) to be consistent with what the reader is familiar with seeing in guideline statements. A Class I recommendation means that the benefits of the AF ablation procedure markedly exceed the risks, and that AF ablation should be performed; a Class IIa recommendation means that the benefits of an AF ablation procedure exceed the risks, and that it is reasonable to perform AF ablation; a Class IIb recommendation means that the benefit of AF ablation is greater or equal to the risks, and that AF ablation may be considered; and a Class III recommendation means that AF ablation is of no proven benefit and is not recommended.

The writing group reviewed and ranked evidence supporting current recommendations with the weight of evidence ranked as Level A if the data were derived from high-quality evidence from more than one randomized clinical trial, meta-analyses of high-quality randomized clinical trials, or one or more randomized clinical trials corroborated by high-quality registry studies. The writing group ranked available evidence as Level B-R when there was moderate-quality evidence from one or more randomized clinical trials, or meta-analyses of moderate-quality randomized clinical trials. Level B-NR was used to denote moderate-quality evidence from one or more well-designed, well-executed nonrandomized studies, observational studies, or registry studies. This designation was also used to denote moderate-quality evidence from meta-analyses of such studies. Evidence was ranked as Level C-LD when the primary source of the recommendation was randomized or nonrandomized observational or registry studies with limitations of design or execution, meta-analyses of such studies, or physiological or mechanistic studies of human subjects. Level C-EO was defined as expert opinion based on the clinical experience of the writing group.

Despite a large number of authors, the participation of several societies and professional organizations, and the attempts of the group to reflect the current knowledge in the field adequately, this document is not intended as a guideline. Rather, the group would like to refer to the current guidelines on AF management for the purpose of guiding overall AF management strategies [5, 6]. This consensus document is specifically focused on catheter and surgical ablation of AF, and summarizes the opinion of the writing group members based on an extensive literature review as well as their own experience. It is directed to all health care professionals who are involved in the care of patients with AF, particularly those who are caring for patients who are undergoing, or are being considered for, catheter or surgical ablation procedures for AF, and those involved in research in the field of AF ablation. This statement is not intended to recommend or promote catheter or surgical ablation of AF. Rather, the ultimate judgment regarding care of a particular patient must be made by the health care provider and the patient in light of all the circumstances presented by that patient.

The main objective of this document is to improve patient care by providing a foundation of knowledge for those involved with catheter ablation of AF. A second major objective is to provide recommendations for designing clinical trials and reporting outcomes of clinical trials of AF ablation. It is recognized that this field continues to evolve rapidly. As this document was being prepared, further clinical trials of catheter and surgical ablation of AF were under way.

## Definitions, mechanisms, and rationale for AF ablation

**Fig. 1:**
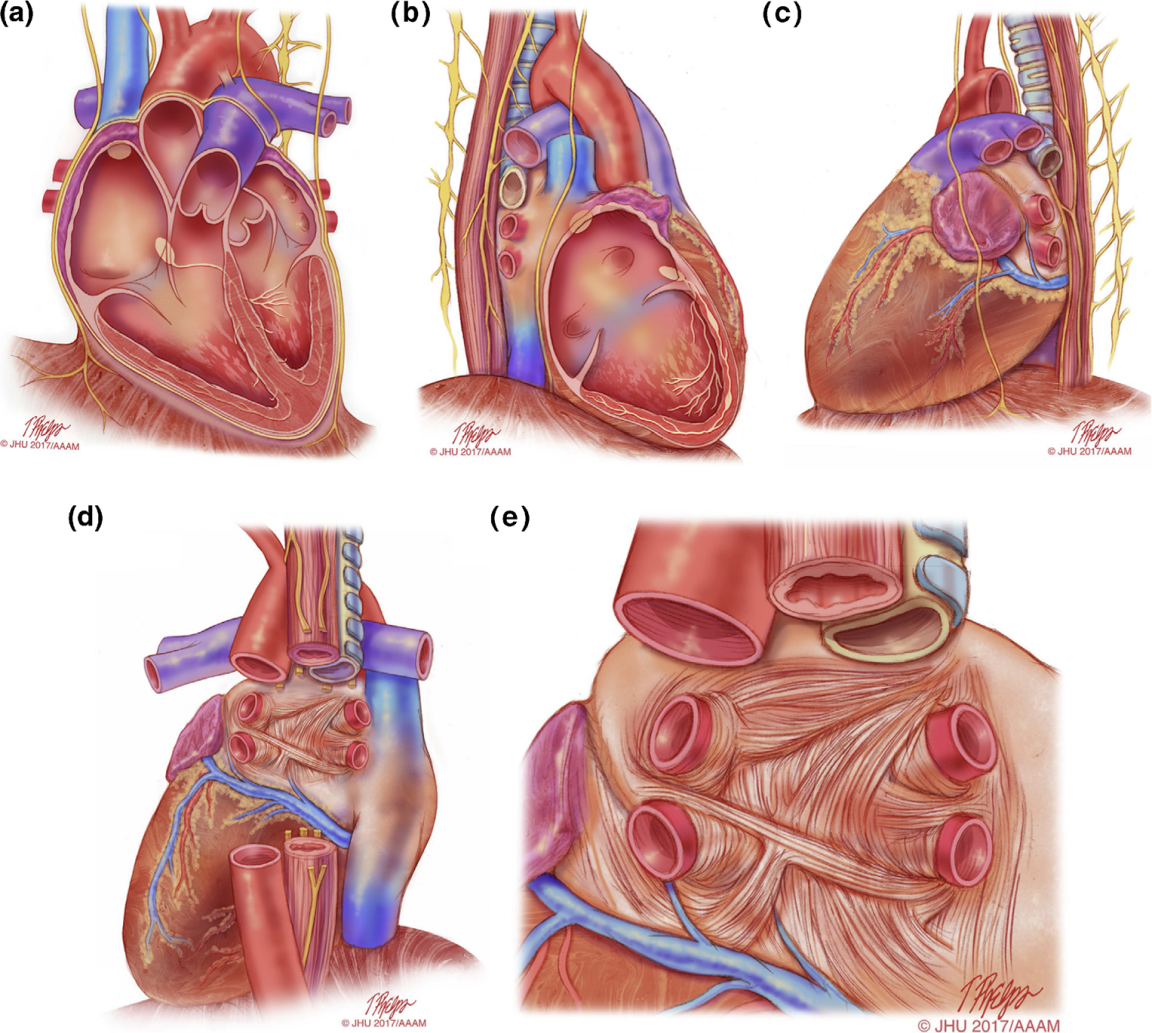
Anatomical drawings of the heart relevant to AF ablation. This series of drawings shows the heart and associated relevant structures from four different perspectives relevant to AF ablation. This drawing includes the phrenic nerves and the esophagus. **a** The heart viewed from the anterior perspective. **b** The heart viewed from the right lateral perspective. **c** The heart viewed from the left lateral perspective. **d** The heart viewed from the posterior perspective. **e** The left atrium viewed from the posterior perspective. *Illustration: Tim Phelps © 2017 Johns Hopkins University, AAM*

**Fig. 2:**
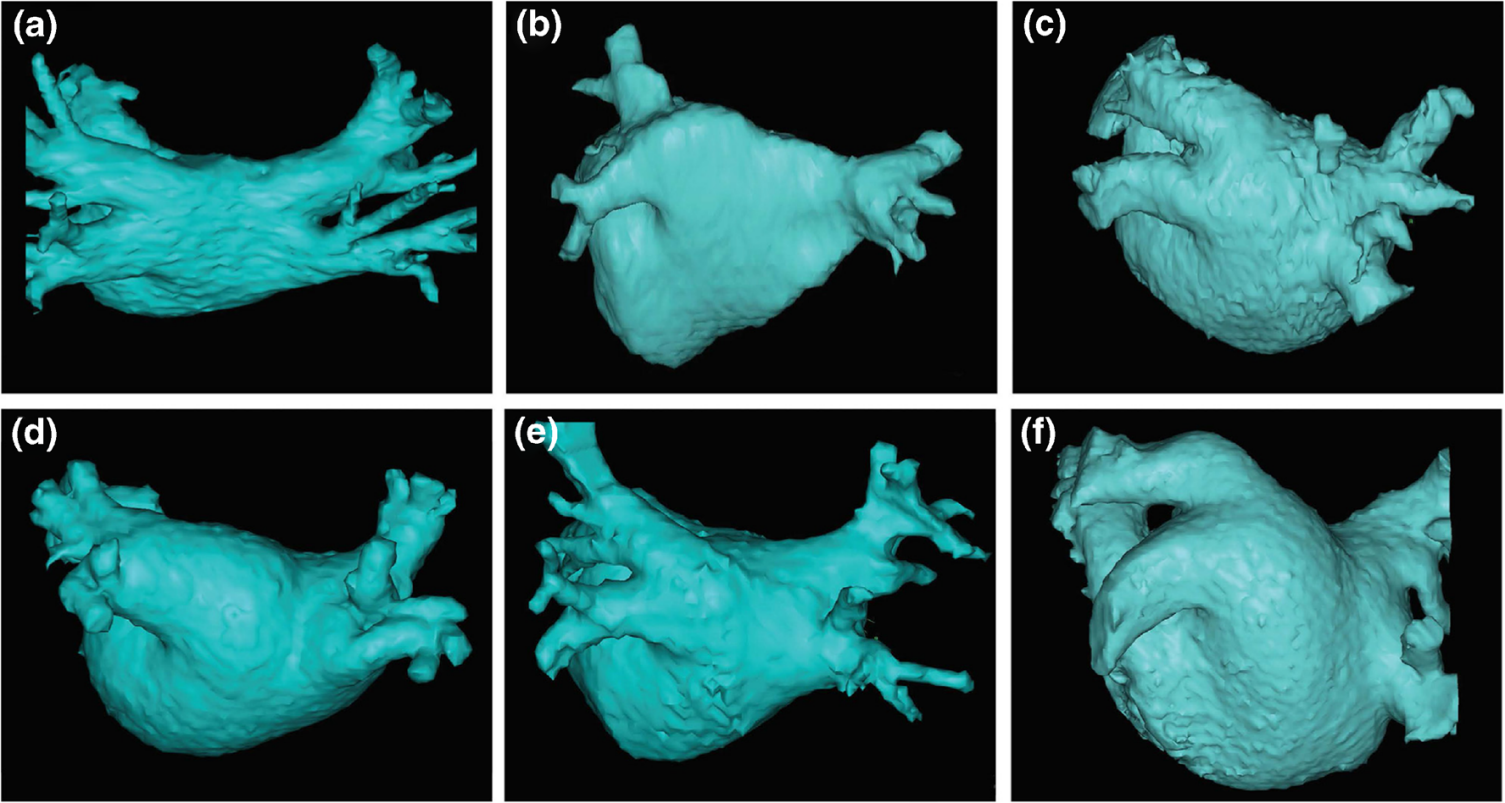
This figure includes six CT or MR images of the left atrium and pulmonary veins viewed from the posterior perspective. Common and uncommon variations in PV anatomy are shown. **a** Standard PV anatomy with 4 distinct PV ostia. **b** Variant PV anatomy with a right common and a left common PV. **c** Variant PV anatomy with a left common PV with a short trunk and an anomolous PV arising from the right posterior left atrial wall. **d** and **e** Variant PV anatomy with a common left PV with a long trunk. **f** Variant PV anatomy with a massive left common PV

**Fig. 3:**
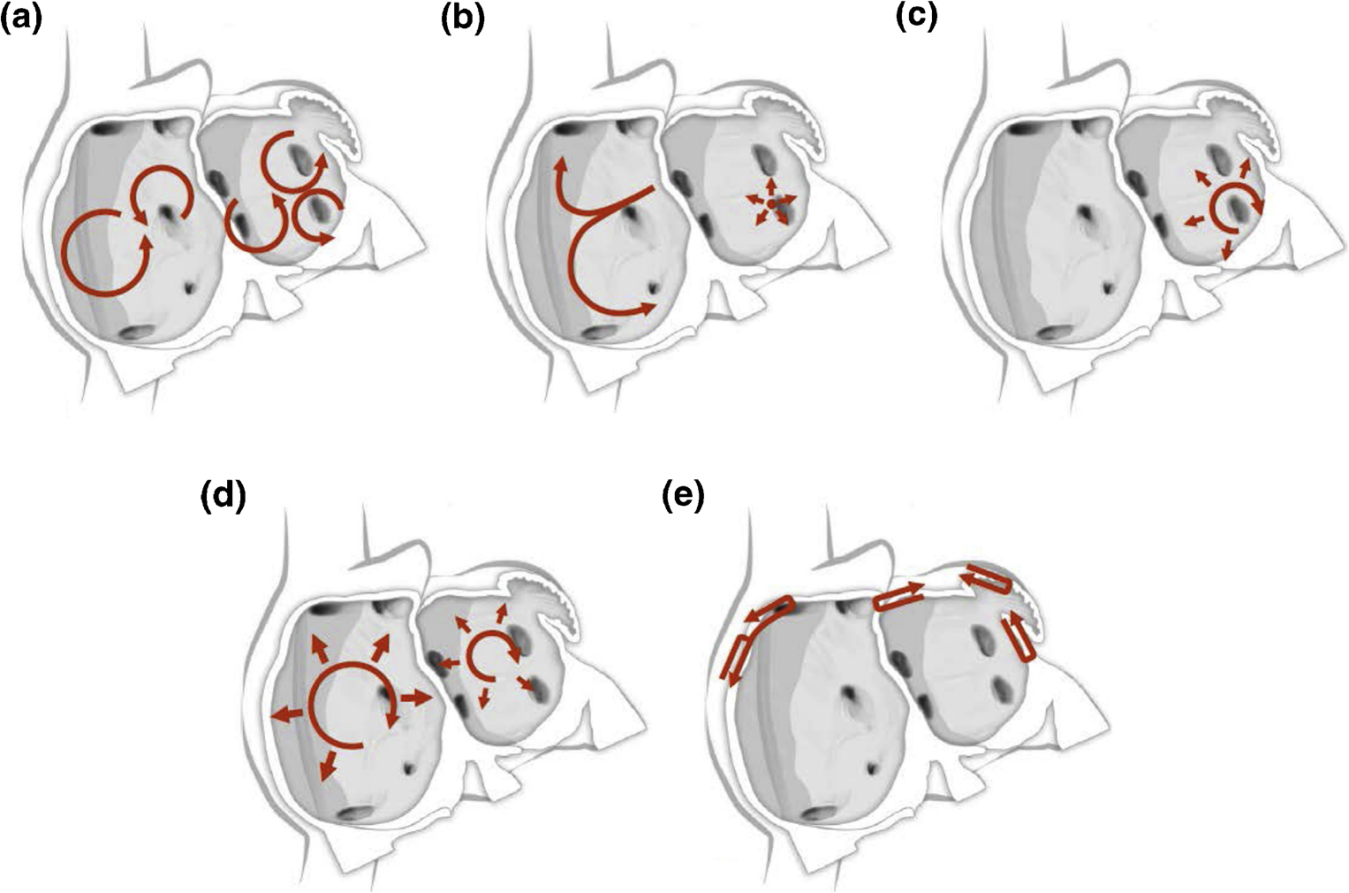
Schematic drawing showing various hypotheses and proposals concerning the mechanisms of atrial fibrillation. **a** Multiple wavelets hypothesis. **b** Rapidly discharging automatic foci. **c** Single reentrant circuit with fibrillatory conduction. **d** Functional reentry resulting from rotors or spiral waves. **e** AF maintenance resulting from dissociation between epicardial and endocardial layers, with mutual interaction producing multiplying activity that maintains the arrhythmia

**Fig. 4:**
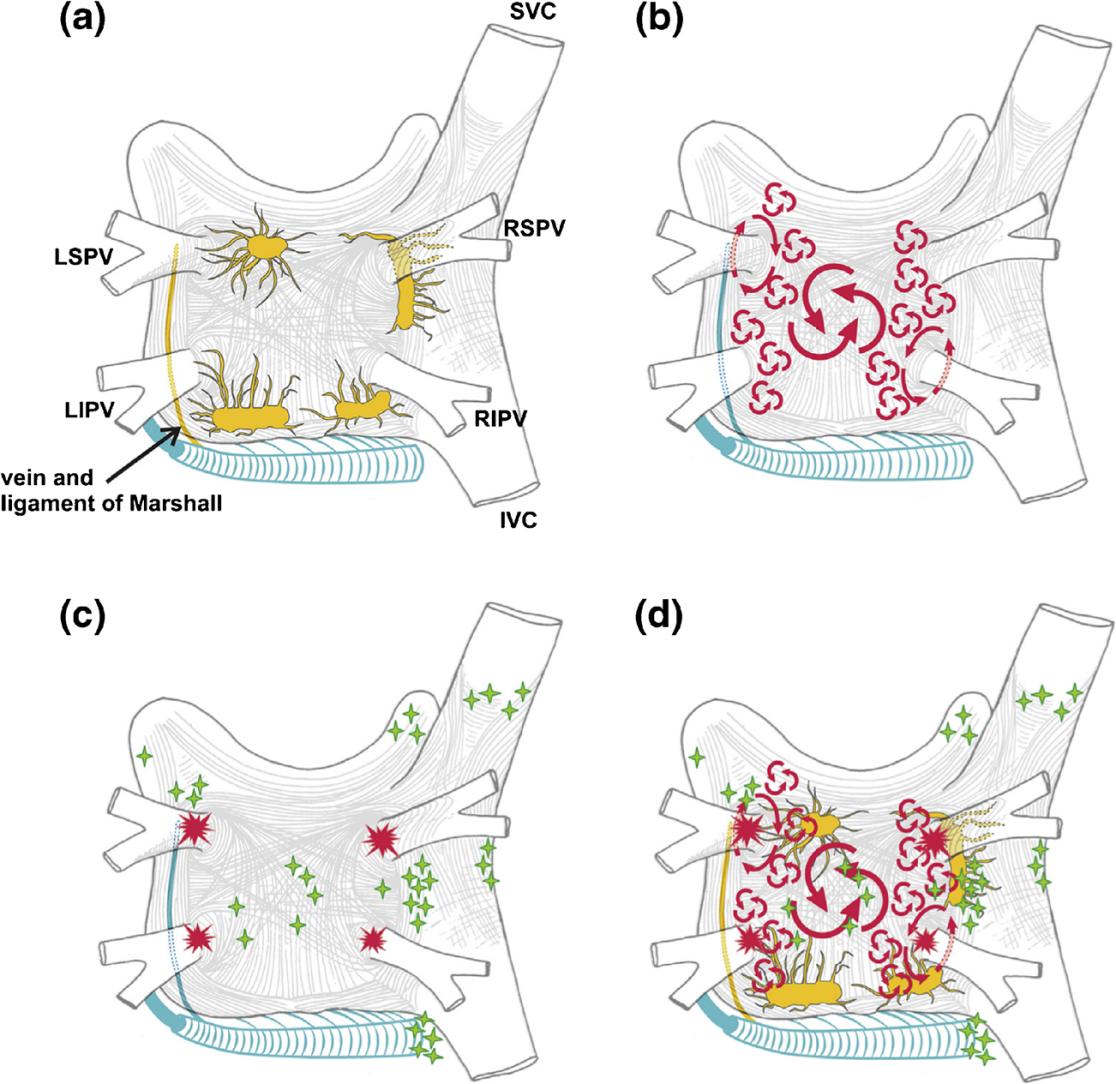
Structure and mechanisms of atrial fibrillation. **a** Schematic drawing of the left and right atria as viewed from the posterior perspective. The extension of muscular fibers onto the PVs can be appreciated. Shown in *yellow* are the five major left atrial autonomic ganglionic plexi (GP) and axons (superior left GP, inferior left GP, anterior right GP, inferior right GP, and ligament of Marshall). Shown in *blue* is the coronary sinus, which is enveloped by muscular fibers that have connections to the atria. Also shown in *blue* is the vein and ligament of Marshall, which travels from the coronary sinus to the region between the left superior PV and the left atrial appendage. **b** The large and small reentrant wavelets that play a role in initiating and sustaining AF. **c** The common locations of PV (*red*) and also the common sites of origin of non-PV triggers (shown in *green*). **d** Composite of the anatomic and arrhythmic mechanisms of AF. Adapted with permission from Calkins et al. Heart Rhythm 2012; 9:632–696.e21 [2]

**Fig. 5:**
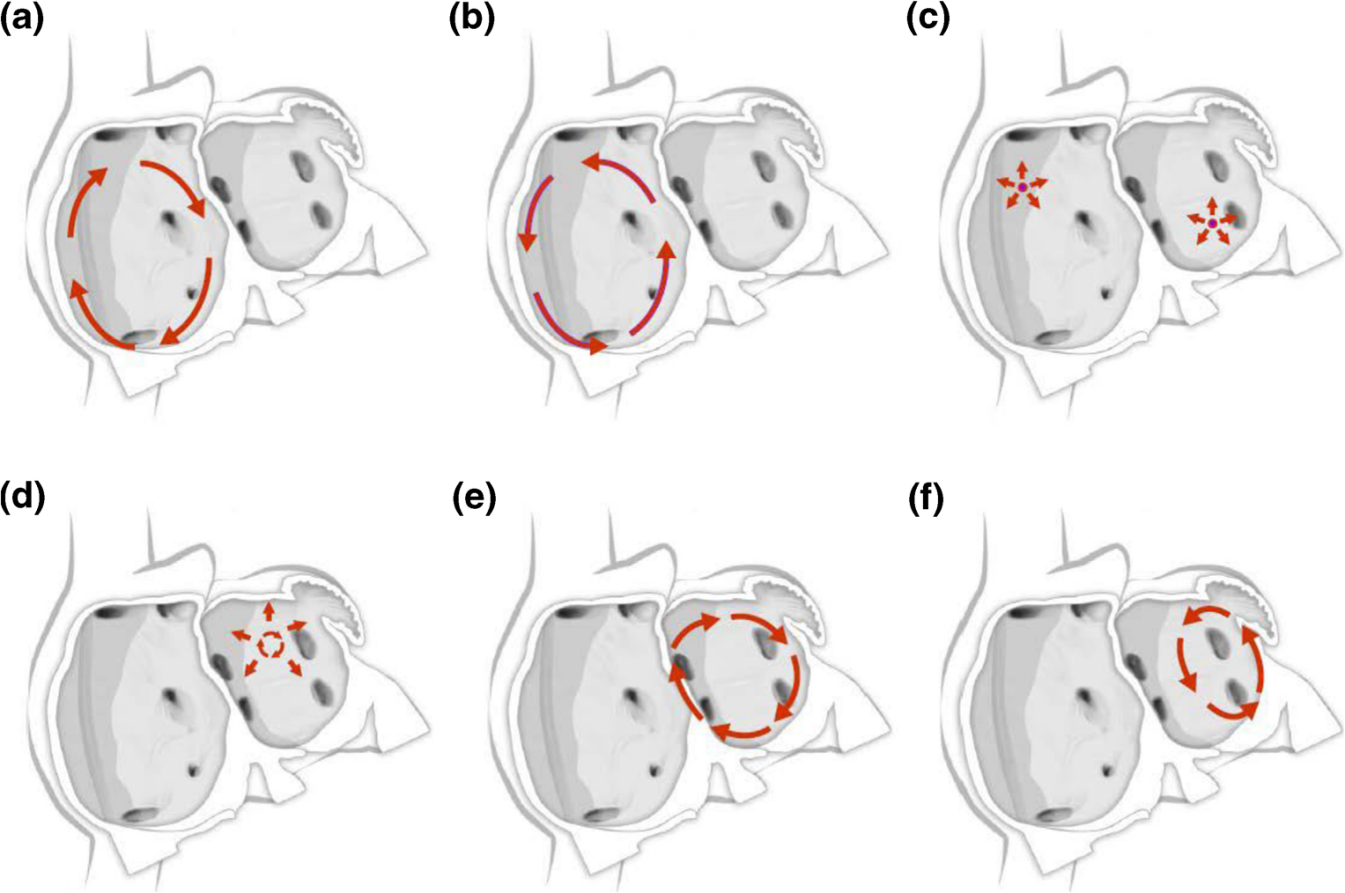
Schematic drawing showing mechanisms of atrial flutter and atrial tachycardia. **a** Isthmus-dependent reverse common (clockwise) atrial flutter. **b** Isthmus-dependent common (counter clockwise) atrial flutter. **c** Focal atrial tachycardia with circumferential spread of activation of the atria (can arise from multiple sites within the left and right atrium). **d** Microreentrant atrial tachycardia with circumferential spread of activation of the atria. **e** Perimitral atrial flutter. **f** Roof-dependent atrial flutter

**Fig. 6:**
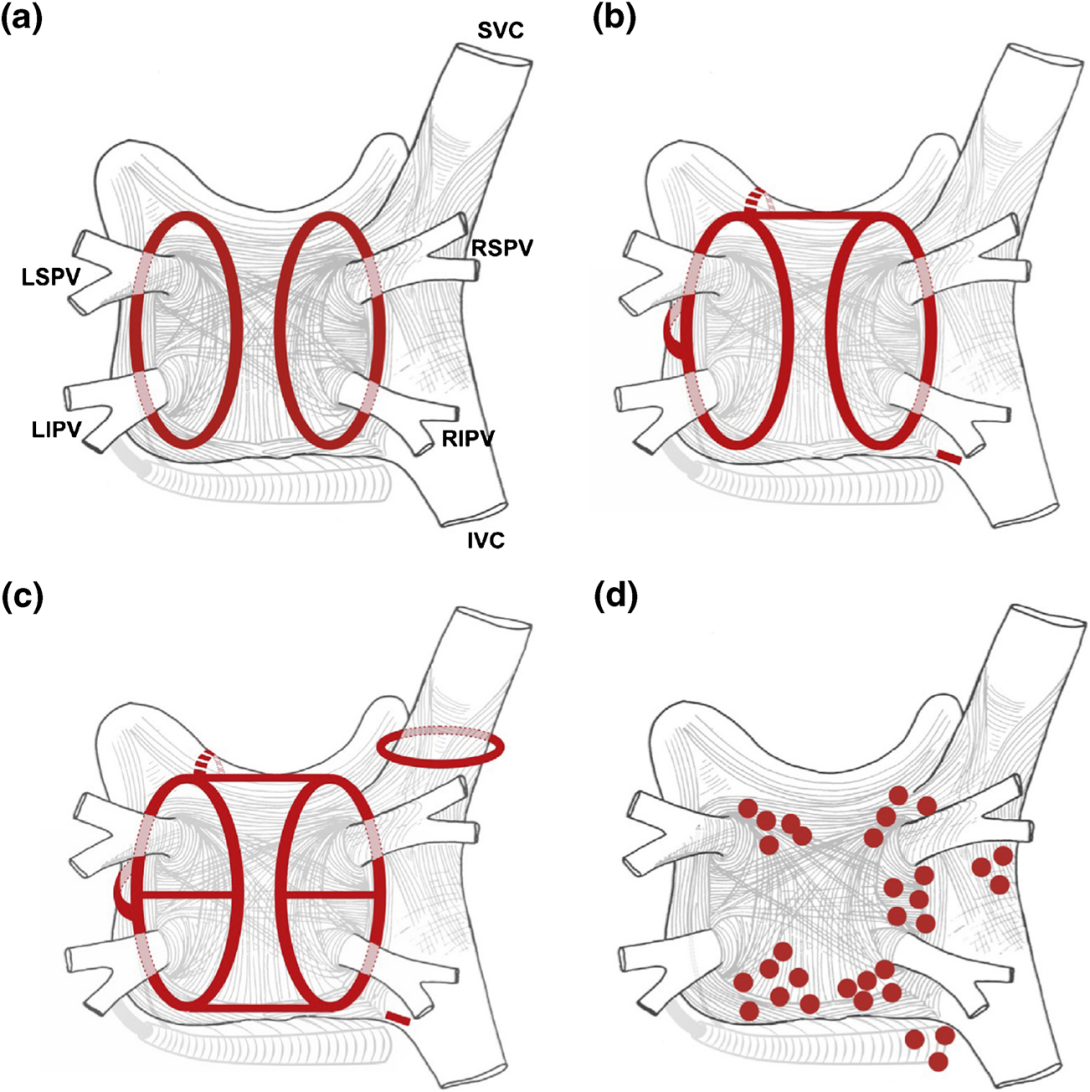
Schematic of common lesion sets employed in AF ablation. **a** The circumferential ablation lesions that are created in a circumferential fashion around the right and the left PVs. The primary endpoint of this ablation strategy is the electrical isolation of the PV musculature. **b** Some of the most common sites of linear ablation lesions. These include a “roof line” connecting the lesions encircling the left and/or right PVs, a “mitral isthmus” line connecting the mitral valve and the lesion encircling the left PVs at the end of the left inferior PV, and an anterior linear lesion connecting either the “roof line” or the left or right circumferential lesion to the mitral annulus anteriorly. A linear lesion created at the cavotricuspid isthmus is also shown. This lesion is generally placed in patients who have experienced cavotricuspid isthmus-dependent atrial flutter clinically or have it induced during EP testing. **c** Similar to 6B, but also shows additional linear ablation lesions between the superior and inferior PVs resulting in a figure of eight lesion sets as well as a posterior inferior line allowing for electrical isolation of the posterior left atrial wall. An encircling lesion of the superior vena cava (SVC) directed at electrical isolation of the SVC is also shown. SVC isolation is performed if focal firing from the SVC can be demonstrated. A subset of operators empirically isolates the SVC. **d** Representative sites for ablation when targeting rotational activity or CFAEs are targeted. Modified with permission from Calkins et al. Heart Rhythm 2012; 9:632–696.e21 [2]

This section of the document provides definitions for use in the diagnosis of AF. This section also provides an in-depth review of the mechanisms of AF and rationale for catheter and surgical AF ablation (Table 1, Figs. 1, 2, 3, 4, 5, and 6).

**Table 1:** Atrial fibrillation definitions

AF episode	An AF episode is defined as AF that is documented by ECG monitoring or intracardiac electrogram monitoring and has a duration of at least 30 s, or if less than 30 s, is present throughout the ECG monitoring tracing. The presence of subsequent episodes of AF requires that sinus rhythm be documented by ECG monitoring between AF episodes.
Chronic AF	Chronic AF has variable definitions and should not be used to describe populations of AF patients undergoing AF ablation.
Early persistent AF	Early persistent AF is defined as AF that is sustained beyond 7 days but is less than 3 months in duration.
Lone AF	Lone AF is a historical descriptor that is potentially confusing and should not be used to describe populations of patients with AF undergoing AF ablation.
Long-standing persistent AF	Long-standing persistent AF is defined as continuous AF of greater than 12 months’ duration.
Paroxysmal AF	Paroxysmal AF is defined as AF that terminates spontaneously or with intervention within 7 days of onset.
Permanent AF	Permanent AF is defined as the presence of AF that is accepted by the patient and physician, and for which no further attempts to restore or maintain sinus rhythm will be undertaken. The term *permanent AF* represents a therapeutic attitude on the part of the patient and physician rather than an inherent pathophysiological attribute of AF. The term *permanent AF* should not be used within the context of a rhythm control strategy with antiarrhythmic drug therapy or AF ablation.
Persistent AF	Persistent AF is defined as continuous AF that is sustained beyond 7 days.
Silent AF	Silent AF is defined as asymptomatic AF diagnosed with an opportune ECG or rhythm strip.

*AF* atrial fibrillation, *ECG* electrocardiogram

## Modifiable risk factors for AF and impact on ablation

Management of patients with AF has traditionally consisted of three main components: (1) anticoagulation for stroke prevention; (2) rate control; and (3) rhythm control. With the emergence of large amounts of data, which have both defined and called attention to the interaction between modifiable risk factors and the development of AF and outcomes of AF management, we believe it is time to include risk factor modification as the fourth pillar of AF management. This section of the document reviews the link between modifiable risk factors and both the development of AF and their impacts on the outcomes of AF ablation.

## Indications

**Fig. 7:**
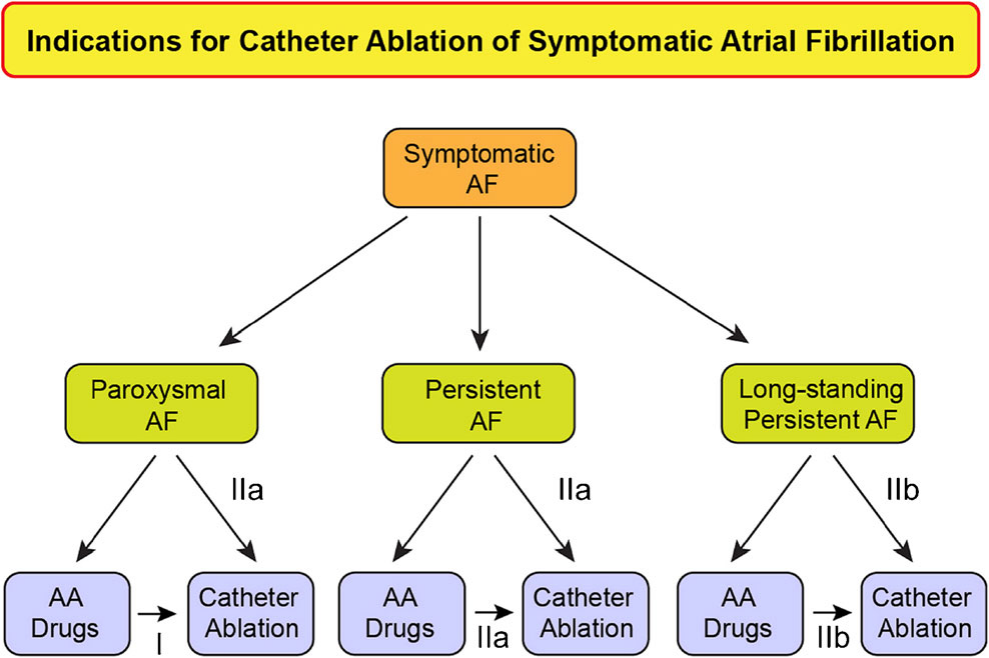
Indications for catheter ablation of symptomatic atrial fibrillation. Shown in this figure are the indications for catheter ablation of symptomatic paroxysmal, persistent, and long-standing persistent AF. The Class for each indication based on whether ablation is performed after failure of antiarrhythmic drug therapy or as first-line therapy is shown. Please refer to Table 2B and the text for the indications for catheter ablation of asymptomatic AF

**Fig. 8:**
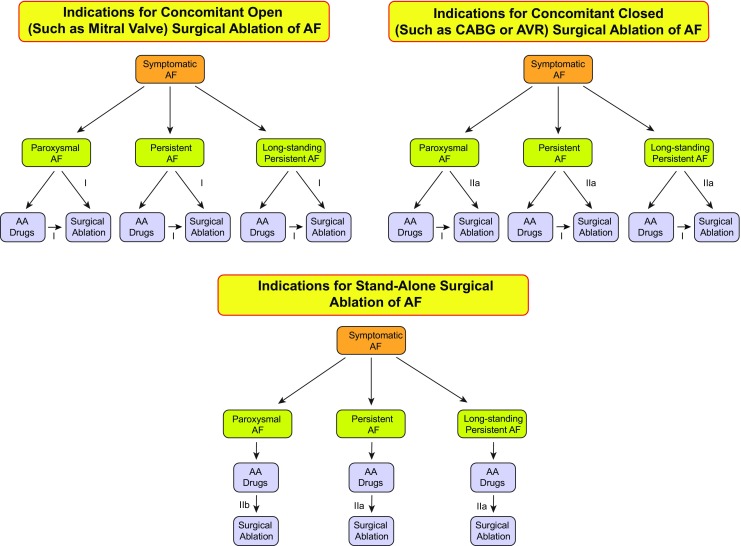
Indications for surgical ablation of atrial fibrillation. Shown in this figure are the indications for surgical ablation of paroxysmal, persistent, and long-standing persistent AF. The Class for each indication based on whether ablation is performed after failure of antiarrhythmic drug therapy or as first-line therapy is shown. The indications for surgical AF ablation are divided into whether the AF ablation procedure is performed concomitantly with an open surgical procedure (such as mitral valve replacement), a closed surgical procedure (such as coronary artery bypass graft surgery), or as a stand-alone surgical AF ablation procedure performed solely for treatment of atrial fibrillation

Shown in Table 2, and summarized in Figs. 7 and 8 of this document, are the Consensus Indications for Catheter and Surgical Ablation of AF. As outlined in the introduction section of this document, these indications are stratified as Class I, Class IIa, Class IIb, and Class III indications. The evidence supporting these indications is provided, as well as a selection of the key references supporting these levels of evidence. In making these recommendations, the writing group considered the body of published literature that has defined the safety and efficacy of catheter and surgical ablation of AF. Also considered in these recommendations is the personal lifetime experience in the field of each of the writing group members. Both the number of clinical trials and the quality of these trials were considered. In considering the class of indications recommended by this writing group, it is important to keep several points in mind. First, these classes of indications only define the indications for catheter and surgical ablation of AF when performed by an electrophysiologist or a surgeon who has received appropriate training and/or who has a certain level of experience and is performing the procedure in an experienced center (Section 11). Catheter and surgical ablation of AF are highly complex procedures, and a careful assessment of the benefit and risk must be considered for each patient. Second, these indications stratify patients based only on the type of AF and whether the procedure is being performed prior to or following a trial of one or more Class I or III antiarrhythmic medications. This document for the first time includes indications for catheter ablation of select asymptomatic patients. As detailed in Section 9, there are many other additional clinical and imaging-based variables that can be used to further define the efficacy and risk of ablation in a given patient. Some of the variables that can be used to define patients in whom a lower success rate or a higher complication rate can be expected include the presence of concomitant heart disease, obesity, sleep apnea, left atrial (LA) size, patient age and frailty, as well as the duration of time the patient has been in continuous AF. Each of these variables needs to be considered when discussing the risks and benefits of AF ablation with a particular patient. In the presence of substantial risk or anticipated difficulty of ablation, it could be more appropriate to use additional antiarrhythmic drug (AAD) options, even if the patient on face value might present with a Class I or IIa indication for ablation. Third, it is important to consider patient preference and values. Some patients are reluctant to consider a major procedure or surgery and have a strong preference for a pharmacological approach. In these patients, trials of antiarrhythmic agents including amiodarone might be preferred to catheter ablation. On the other hand, some patients prefer a nonpharmacological approach. Fourth, it is important to recognize that some patients early in the course of their AF journey might have only infrequent episodes for many years and/or could have AF that is responsive to well-tolerated AAD therapy. And finally, it is important to bear in mind that a decision to perform catheter or surgical AF ablation should only be made after a patient carefully considers the risks, benefits, and alternatives to the procedure.

**Table 2:** Indications for catheter (A and B) and surgical (C, D, and E) ablation of atrial fibrillation

	Recommendation	Class	LOE	References
Indications for catheter ablation of atrial fibrillation				
A. Indications for catheter ablation of atrial fibrillation				
Symptomatic AF refractory or intolerant to at least one Class I or III antiarrhythmic medication	Paroxysmal: Catheter ablation is recommended.	I	A	[7–18]
	Persistent: Catheter ablation is reasonable.	IIa	B-NR	[8, 16–26]
	Long-standing persistent: Catheter ablation may be considered.	IIb	C-LD	[8, 16–26]
Symptomatic AF prior to initiation of antiarrhythmic therapy with a Class I or III antiarrhythmic medication	Paroxysmal: Catheter ablation is reasonable.	IIa	B-R	[27–35]
	Persistent: Catheter ablation is reasonable.	IIa	C-EO	
	Long-standing persistent: Catheter ablation may be considered.	IIb	C-EO	
B. Indications for catheter atrial fibrillation ablation in populations of patients not well represented in clinical trials				
Congestive heart failure	It is reasonable to use similar indications for AF ablation in selected patients with heart failure as in patients without heart failure.	IIa	B-R	[36–52]
Older patients (>75 years of age)	It is reasonable to use similar indications for AF ablation in selected older patients with AF as in younger patients.	IIa	B-NR	[53–59]
Hypertrophic cardiomyopathy	It is reasonable to use similar indications for AF ablation in selected patients with HCM as in patients without HCM.	IIa	B-NR	[60–62]
Young patients (<45 years of age)	It is reasonable to use similar indications for AF ablation in young patients with AF (<45 years of age) as in older patients.	IIa	B-NR	[63, 64]
Tachy-brady syndrome	It is reasonable to offer AF ablation as an alternative to pacemaker implantation in patients with tachy-brady syndrome.	IIa	B-NR	[33–35]
Athletes with AF	It is reasonable to offer high-level athletes AF as first-line therapy due to the negative effects of medications on athletic performance.	IIa	C-LD	[27, 28, 65]
Asymptomatic AF^∗∗^	Paroxysmal: Catheter ablation may be considered in select patients.^∗∗^	IIb	C-EO	[66, 67]
	Persistent: Catheter ablation may be considered in select patients.	IIb	C-EO	[68]
Indications for surgical ablation of atrial fibrillation				
C. Indications for concomitant open (such as mitral valve) surgical ablation of atrial fibrillation				
Symptomatic AF refractory or intolerant to at least one Class I or III antiarrhythmic medication	Paroxysmal: Surgical ablation is recommended.	I	B-NR	[69–82]
	Persistent: Surgical ablation is recommended.	I	B-NR	[69–82]
	Long-standing persistent: Surgical ablation is recommended.	I	B-NR	[69–82]
Symptomatic AF prior to initiation of antiarrhythmic therapy with a Class I or III antiarrhythmic medication	Paroxysmal: Surgical ablation is recommended.	I	B-NR	[69–82]
	Persistent: Surgical ablation is recommended.	I	B-NR	[69–82]
	Long-standing persistent: Surgical ablation is recommended.	I	B-NR	[69–82]
D. Indications for concomitant closed (such as CABG and AVR) surgical ablation of atrial fibrillation				
Symptomatic AF refractory or intolerant to at least one Class I or III antiarrhythmic medication	Paroxysmal: Surgical ablation is recommended.	I	B-NR	[83–88]
	Persistent: Surgical ablation is recommended.	I	B-NR	[83–88]
	Long-standing persistent: Surgical ablation is recommended.	I	B-NR	[83–88]
Symptomatic AF prior to initiation of antiarrhythmic therapy with a Class I or III antiarrhythmic medication	Paroxysmal: Surgical ablation is reasonable.	IIa	B-NR	[83–88]
	Persistent: Surgical ablation is reasonable.	IIa	B-NR	[83–88]
	Long-standing persistent: Surgical ablation is reasonable.	IIa	B-NR	[83–88]
E. Indications for stand-alone and hybrid surgical ablation of atrial fibrillation				
Symptomatic AF refractory or intolerant to at least one Class I or III antiarrhythmic medication	Paroxysmal: Stand-alone surgical ablation can be considered for patients who have failed one or more attempts at catheter ablation and also for those who are intolerant or refractory to antiarrhythmic drug therapy and prefer a surgical approach, after review of the relative safety and efficacy of catheter ablation versus a stand-alone surgical approach.	IIb	B-NR	[83–85, 89–103]
	Persistent: Stand-alone surgical ablation is reasonable for patients who have failed one or more attempts at catheter ablation and also for those patients who prefer a surgical approach after review of the relative safety and efficacy of catheter ablation versus a stand-alone surgical approach.	IIa	B-NR	[83–85, 89–103]
	Long-standing persistent: Stand-alone surgical ablation is reasonable for patients who have failed one or more attempts at catheter ablation and also for those patients who prefer a surgical approach after review of the relative safety and efficacy of catheter ablation versus a stand-alone surgical approach.	IIa	B-NR	[83–85, 89–103]
	It might be reasonable to apply the indications for stand-alone surgical ablation described above to patients being considered for hybrid surgical AF ablation.	IIb	C-EO	[103–108]

*AF* atrial fibrillation, *LOE* Level of Evidence, *HCM* hypertrophic cardiomyopathy

^∗∗^A decision to perform AF ablation in an asymptomatic patient requires additional discussion with the patient because the potential benefits of the procedure for the patient without symptoms are uncertain

## Strategies, techniques, and endpoints

The writing group recommendations for techniques to be used for ablation of persistent and long-standing persistent AF (Table 3), adjunctive ablation strategies, nonablative strategies to improve outcomes of AF ablation, and endpoints for ablation of paroxysmal, persistent, and long-standing persistent AF are covered in this section. A schematic overview of common lesion sets created during an AF ablation procedure is shown in Fig. 6.

**Table 3:** Atrial fibrillation ablation: strategies, techniques, and endpoints

	Recommendation	Class	LOE	References
PV isolation by catheter ablation	Electrical isolation of the PVs is recommended during all AF ablation procedures.	I	A	[7–16, 19–26, 109]
	Achievement of electrical isolation requires, at a minimum, assessment and demonstration of entrance block into the PV.	I	B-R	[7–16, 19–26, 109]
	Monitoring for PV reconnection for 20 min following initial PV isolation is reasonable.	IIa	B-R	[9, 110–120]
	Administration of adenosine 20 min following initial PV isolation using RF energy with reablation if PV reconnection might be considered.	IIb	B-R	[109, 111–114, 120–128]
	Use of a pace-capture (pacing along the ablation line) ablation strategy may be considered.	IIb	B-R	[129–133]
	Demonstration of exit block may be considered.	IIb	B-NR	[134–139]
Ablation strategies to be considered for use in conjunction with PV isolation	If a patient has a history of typical atrial flutter or typical atrial flutter is induced at the time of AF ablation, delivery of a cavotricuspid isthmus linear lesion is recommended.	I	B-R	[140–143]
	If linear ablation lesions are applied, operators should use mapping and pacing maneuvers to assess for line completeness.	I	C-LD	[19, 141–149]
	If a reproducible focal trigger that initiates AF is identified outside the PV ostia at the time of an AF ablation procedure, ablation of the focal trigger should be considered.	IIa	C-LD	[150–161]
	When performing AF ablation with a force-sensing RF ablation catheter, a minimal targeted contact force of 5 to 10 g is reasonable.	IIa	C-LD	[13, 14, 128, 162–178]
	Posterior wall isolation might be considered for initial or repeat ablation of persistent or long-standing persistent AF.	IIb	C-LD	[21, 179–185]
	Administration of high-dose isoproterenol to screen for and then ablate non-PV triggers may be considered during initial or repeat AF ablation procedures in patients with paroxysmal, persistent, or long-standing persistent AF.	IIb	C-LD	[150–161]
	DF-based ablation strategy is of unknown usefulness for AF ablation.	IIb	C-LD	[186–193]
	The usefulness of creating linear ablation lesions in the right or left atrium as an initial or repeat ablation strategy for persistent or long-standing persistent AF is not well established.	IIb	B-NR	[19, 20, 142, 145–149, 194–201]
	The usefulness of linear ablation lesions in the absence of macroreentrant atrial flutter is not well established.	IIb	C-LD	[19, 20, 142, 145–149, 194–201]
	The usefulness of mapping and ablation of areas of abnormal myocardial tissue identified with voltage mapping or MRI as an initial or repeat ablation strategy for persistent or long-standing persistent AF is not well established.	IIb	B-R	[179, 202–211]
	The usefulness of ablation of complex fractionated atrial electrograms as an initial or repeat ablation strategy for persistent and long-standing persistent AF is not well established.	IIb	B-R	[19, 20, 195–197, 212–220]
	The usefulness of ablation of rotational activity as an initial or repeat ablation strategy for persistent and long-standing persistent AF is not well established.	IIb	B-NR	[221–241]
	The usefulness of ablation of autonomic ganglia as an initial or repeat ablation strategy for paroxysmal, persistent, and long-standing persistent AF is not well established.	IIb	B-NR	[19, 89, 242–259]
Nonablation strategies to improve outcomes	Weight loss can be useful for patients with AF, including those who are being evaluated to undergo an AF ablation procedure, as part of a comprehensive risk factor management strategy.	IIa	B-R	[260–288]
	It is reasonable to consider a patient's BMI when discussing the risks, benefits, and outcomes of AF ablation with a patient being evaluated for an AF ablation procedure.	IIa	B-R	[260–288]
	It is reasonable to screen for signs and symptoms of sleep apnea when evaluating a patient for an AF ablation procedure and to recommend a sleep evaluation if sleep apnea is suspected.	IIa	B-R	[270, 276–278, 289–307]
	Treatment of sleep apnea can be useful for patients with AF, including those who are being evaluated to undergo an AF ablation procedure.	IIa	B-R	[270, 276–278, 289–307]
	The usefulness of discontinuation of antiarrhythmic drug therapy prior to AF ablation in an effort to improve long-term outcomes is unclear.	IIb	C-LD	[308–312]
	The usefulness of initiation or continuation of antiarrhythmic drug therapy during the postablation healing phase in an effort to improve long-term outcomes is unclear.	IIb	C-LD	[308–312]
Strategies to reduce the risks of AF ablation	Careful identification of the PV ostia is mandatory to avoid ablation within the PVs.	I	B-NR	[313–335]
	It is recommended that RF power be reduced when creating lesions along the posterior wall near the esophagus.	I	C-LD	[68, 336–365]
	It is reasonable to use an esophageal temperature probe during AF ablation procedures to monitor esophageal temperature and help guide energy delivery.	IIa	C-EO	[68, 336, 345, 365]

*AF* atrial fibrillation, *LOE* Level of Evidence, *PV* pulmonary vein, *RF* radiofrequency, *MRI* magnetic resonance imaging, *BMI* body mass index

## Technology and tools

**Fig. 9:**
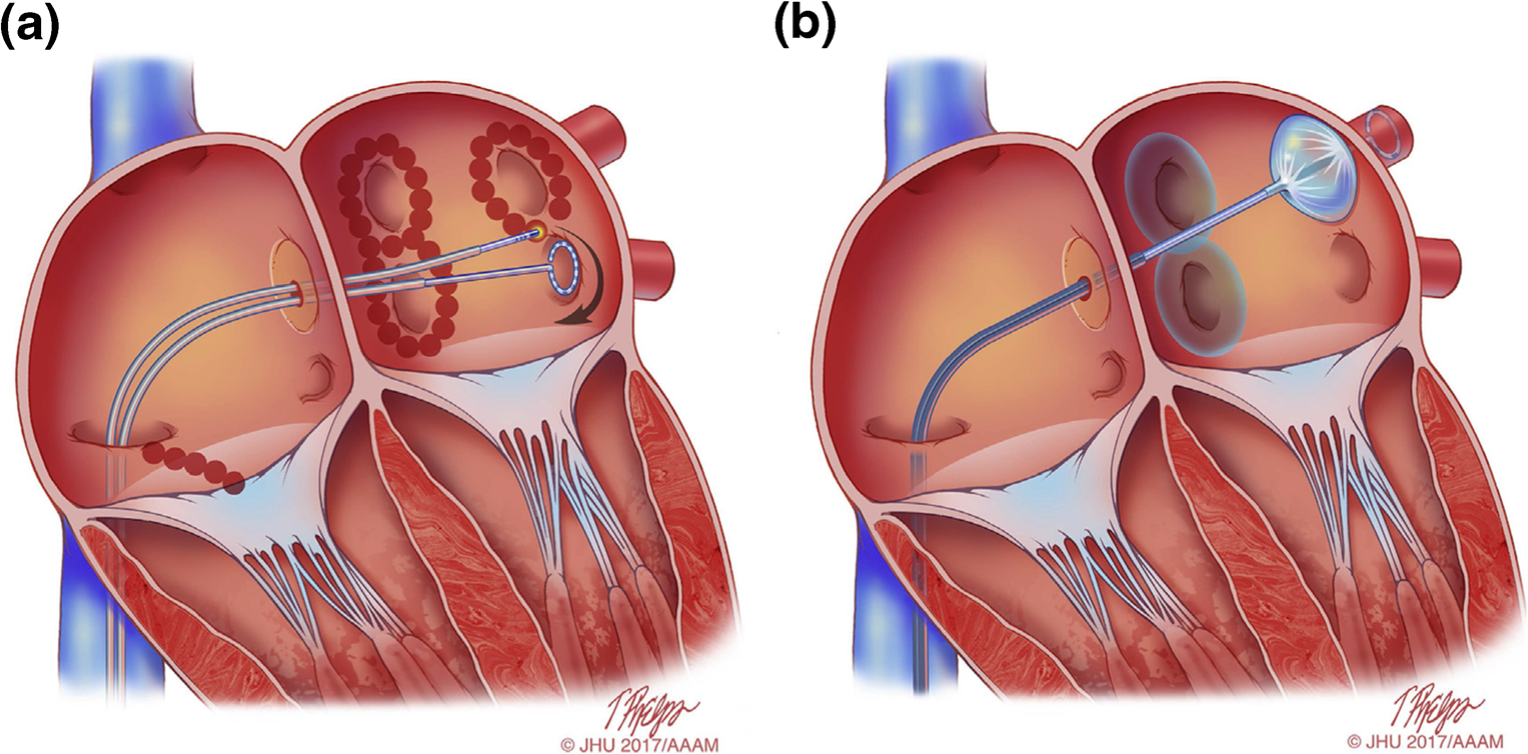
Schematic drawing showing catheter ablation of atrial fibrillation using either RF energy or cryoballoon AF ablation. **a** Shows a typical wide area lesion set created using RF energy. Ablation lesions are delivered in a figure of eight pattern around the left and right PV veins. Also shown is a linear cavotricuspid isthmus lesion created for ablation of typical atrial flutter in a patient with a prior history of typical atrial flutter or inducible isthmus-dependent typical atrial flutter at the time of ablation. A multielectrode circular mapping catheter is positioned in the left inferior PV. **b** Shows an ablation procedure using the cryoballoon system. Ablation lesions have been created surrounding the right PVs, and the cryoballoon ablation catheter is positioned in the left superior PV. A through the lumen multielectrode circular mapping catheter is positioned in the left superior PV. *Illustration: Tim Phelps © 2017 Johns Hopkins University, AAM*

This section of the consensus statement provides an update on many of the technologies and tools that are employed for AF ablation procedures. It is important to recognize that this is not a comprehensive listing and that new technologies, tools, and approaches are being developed. It is also important to recognize that radiofrequency (RF) energy is the dominant energy source available for ablation of typical and atypical atrial flutter (AFL). Although cryoablation is a commonly employed tool for AF ablation, it is not well suited for ablation of typical or atypical AFL. Other energy sources and tools are available in some parts of the world and/or are in various stages of development and/or clinical investigation. Shown in Fig. 9 are schematic drawings of AF ablation using point-by-point RF energy (Fig. 9a) and AF ablation using the cryoballoon (CB) system (Fig. 9b).

## Technical aspects of ablation to maximize safety and anticoagulation

Anticoagulation strategies pre-, during, and postcatheter ablation of AF (Table 4); signs and symptoms of complications that can occur within the first several months following ablation (Table 5); anesthesia or sedation during ablation; and approaches to minimize risk of an atrial esophageal fistula are discussed in this section.

**Table 4:** Anticoagulation strategies: pre-, during, and postcatheter ablation of AF

	Recommendation	Class	LOE	References
Preablation	For patients undergoing AF catheter ablation who have been therapeutically anticoagulated with warfarin or dabigatran, performance of the ablation procedure without interruption of warfarin or dabigatran is recommended.	I	A	[366–373]
	For patients undergoing AF catheter ablation who have been therapeutically anticoagulated with rivaroxaban, performance of the ablation procedure without interruption of rivaroxaban is recommended.	I	B-R	[374]
	For patients undergoing AF catheter ablation who have been therapeutically anticoagulated with a NOAC other than dabigatran or rivaroxaban, performance of the ablation procedure without withholding a NOAC dose is reasonable.	IIa	B-NR	[375]
	Anticoagulation guidelines that pertain to cardioversion of AF should be adhered to in patients who present for an AF catheter ablation procedure.	I	B-NR	[5, 6]
	For patients anticoagulated with a NOAC prior to AF catheter ablation, it is reasonable to hold one to two doses of the NOAC prior to AF ablation with reinitiation postablation.	IIa	B-NR	[372, 376–380]
	Performance of a TEE in patients who are in AF on presentation for AF catheter ablation and who have been receiving anticoagulation therapeutically for 3 weeks or longer is reasonable.	IIa	C-EO	[5, 6]
	Performance of a TEE in patients who present for ablation in sinus rhythm and who have not been anticoagulated prior to catheter ablation is reasonable.	IIa	C-EO	[5, 6]
	Use of intracardiac echocardiography to screen for atrial thrombi in patients who cannot undergo TEE may be considered.	IIb	C-EO	[381–386]
During ablation	Heparin should be administered prior to or immediately following transseptal puncture during AF catheter ablation procedures and adjusted to achieve and maintain an ACT of at least 300 s.	I	B-NR	[369, 380–382, 387–393]
	Administration of protamine following AF catheter ablation to reverse heparin is reasonable.	IIa	B-NR	[394]
Postablation	In patients who are not therapeutically anticoagulated prior to catheter ablation of AF and in whom warfarin will be used for anticoagulation postablation, low molecular weight heparin or intravenous heparin should be used as a bridge for initiation of systemic anticoagulation with warfarin following AF ablation.^∗^	I	C-EO	
	Systemic anticoagulation with warfarin^∗^or a NOAC is recommended for at least 2 months postcatheter ablation of AF.	I	C-EO	[1, 2]
	Adherence to AF anticoagulation guidelines is recommended for patients who have undergone an AF ablation procedure, regardless of the apparent success or failure of the procedure.	I	C-EO	[5, 6]
	Decisions regarding continuation of systemic anticoagulation more than 2 months post ablation should be based on the patient's stroke risk profile and not on the perceived success or failure of the ablation procedure.	I	C-EO	[5, 6]
	In patients who have not been anticoagulated prior to catheter ablation of AF or in whom anticoagulation with a NOAC or warfarin has been interrupted prior to ablation, administration of a NOAC 3 to 5 h after achievement of hemostasis is reasonable postablation.	IIa	C-EO	[372, 376–380]
	Patients in whom discontinuation of anticoagulation is being considered based on patient values and preferences should consider undergoing continuous or frequent ECG monitoring to screen for AF recurrence.	IIb	C-EO	

*AF* atrial fibrillation, *LOE* Level of Evidence, *NOAC* novel oral anticoagulant, *TEE* transesophageal electrocardiogram, *ACT* activated clotting time

^∗^Time in therapeutic range (TTR) should be > 65% – 70% on warfarin

**Table 5:** Signs and symptoms following AF ablation

	Differential	Suggested evaluation
Signs and symptoms of complications within a month postablation		
Back pain	Musculoskeletal, retroperitoneal hematoma	Physical exam, CT imaging
Chest pain	Pericarditis, pericardial effusion, coronary stenosis (ablation related), pulmonary vein stenosis, musculoskeletal (after cardioversion), worsening reflux	Physical exam, chest X-ray, ECG, echocardiogram, stress test, cardiac catheterization, chest CT
Cough	Infectious process, bronchial irritation (mechanical, cryoballoon), pulmonary vein stenosis	Physical exam, chest X-ray, chest CT
Dysphagia	Esophageal irritation (related to transesophageal echocardiography), atrioesophageal fistula	Physical exam, chest CT or MRI
Early satiety, nausea	Gastric denervation	Physical exam, gastric emptying study
Fever	Infectious process, pericarditis, atrioesophageal fistula	Physical exam, chest X-ray, chest CT, urinalysis, laboratory blood work
Fever, dysphagia, neurological symptoms	Atrial esophageal fistula	Physical exam, laboratory blood work, chest CT or MRI; avoid endoscopy with air insufflation
Groin pain at site of access	Pseudoaneurysm, AV fistula, hematoma	Ultrasound of the groin, laboratory blood work; consider CT scan if ultrasound negative
Headache	Migraine (related to anesthesia or transseptal access, hemorrhagic stroke), effect of general anesthetic	Physical exam, brain imaging (MRI)
Hypotension	Pericardial effusion/tamponade, bleeding, sepsis, persistent vagal reaction	Echocardiography, laboratory blood work
Hemoptysis	PV stenosis or occlusion, pneumonia	Chest X-ray, chest CT or MR scan, VQ scan
Neurological symptoms	Cerebral embolic event, atrial esophageal fistula	Physical exam, brain imaging, chest CT or MRI
Shortness of breath	Volume overload, pneumonia, pulmonary vein stenosis, phrenic nerve injury	Physical exam, chest X-ray, chest CT, laboratory blood work
Signs and symptoms of complications more than a month postablation		
Fever, dysphagia, neurological symptoms	Atrial esophageal fistula	Physical exam, laboratory blood work, chest CT or MRI; avoid endoscopy with air insufflation
Persistent cough, atypical chest pain	Infectious process, pulmonary vein stenosis	Physical exam, laboratory blood work, chest X-ray, chest CT or MRI
Neurological symptoms	Cerebral embolic event, atrial esophageal fistula	Physical exam, brain imaging, chest CT or MRI
Hemoptysis	PV stenosis or occlusion, pneumonia	CT scan, VQ scan

*AF* atrial fibrillation, *ECG* electrocardiogram, *CT* computed tomography, *MRI* magnetic resonance imaging, *VQ* ventilation-perfusion

## Follow-up considerations

AF ablation is an invasive procedure that entails risks, most of which are present during the acute procedural period. However, complications can also occur in the weeks or months following ablation. Recognizing common symptoms after AF ablation and distinguishing those that require urgent evaluation and referral to an electrophysiologist is an important part of follow-up after AF ablation. The success of AF ablation is based in large part on freedom from AF recurrence based on ECG monitoring. Arrhythmia monitoring can be performed with the use of noncontinuous or continuous ECG monitoring tools (Table 6). This section also discusses the important topics of AAD and non-AAD use prior to and following AF ablation, the role of cardioversion, as well as the indications for and timing of repeat AF ablation procedures.

**Table 6:** Types of ambulatory cardiac monitoring devices

Type of recorder	Typical monitoring duration	Continuous recording	Event recording	Auto trigger	Unique features
Holter monitor	24–48 h, approximately 7–30 days	Yes	Yes	N/A	Short term, provides quantitative data on arrhythmia burden
Patch monitor	1–3 weeks	Yes	Yes	N/A	Intermediate term, can provide continuous data for up to several weeks; improved patient compliance without lead wires
External loop recorder	1 month	Yes	Yes	Variable	Good correlation between symptoms and even brief arrhythmias
External nonloop recorder	Months	No	Yes	No	May be used long term and intermittently; will not capture very brief episodes
Smartphone monitor	Indefinite	No	Yes	No	Provides inexpensive long-term intermittent monitoring; dependent on patient compliance; requires a smartphone
Mobile cardiac telemetry	30 days	Yes	Yes	Yes	Real time central monitoring and alarms; relatively expensive
Implantable loop recorder	Up to 3 years	Yes	Yes	Yes	Improved patient compliance for long-term use; not able to detect 30-s episodes of AF due to detection algorithm; presence of AF needs to be confirmed by EGM review because specificity of detection algorithm is imperfect; expensive
Pacemakers or ICDs with atrial leads	Indefinite	Yes	Yes	Yes	Excellent AF documentation of burden and trends; presence of AF needs to be confirmed by electrogram tracing review because specificity of detection algorithms is imperfect; expensive
Wearable multisensor ECG monitors	Indefinite	Yes	Yes	Yes	ECG 3 leads, temp, heart rate, HRV, activity tracking, respiratory rate, galvanic skin response

*AF* atrial fibrillation, *ICD* implantable cardioverter defibrillator, *ECG* electrocardiogram, *HRV* heart rate variability

## Outcomes and efficacy

This section provides a comprehensive review of the outcomes of catheter ablation of AF. Table 7 summarizes the main findings of the most important clinical trials in this field. Outcomes of AF ablation in subsets of patients not well represented in these trials are reviewed. Outcomes for specific ablation systems and strategies (CB ablation, rotational activity ablation, and laser balloon ablation) are also reviewed.

**Table 7:** Selected clinical trials of catheter ablation of atrial fibrillation and/or for FDA approval

Trial	Year	Type	*N*	AF type	Ablation strategy	Initial time frame	Effectiveness endpoint	Ablation success	Drug/ Control success	*P* value for success	Ablation complications	Drug/Control complications	Comments
Clinical Trials Performed for FDA Approval													
JAMA 2010; 303: 333-340 (ThermoCool AF) [14]	2010	Randomized to RF ablation or AAD, multicenter	167	Paroxysmal	PVI, optional CFAEs and lines	12 months	Freedom from symptomatic paroxysmal atrial fibrillation, acute procedural failure, or changes in specified drug regimen	66%	16%	<0.001	4.9%	8.8%	FDA approval received
JACC 2013; 61: 1713-1723 (STOP AF) [9]	2013	Randomized to cryoballoon ablation or AAD, multicenter	245	Paroxysmal	PVI	12 months	Freedom from any detectable AF, use of nonstudy AAD, or nonprotocol intervention for AF	70%	7%	<0.001	3.1%	NA	FDA approval received
Heart Rhythm 2014; 11: 202-209 (TTOP) [22]	2014	Randomized to phased RF ablation or AAD/cardioversion, multicenter	210	Persistent	PVI + CFAEs	6 months	Acute procedural success, ≥90% reduction in AF burden, off AAD	56%	26%	<0.001	12.3%	NA	Not FDA approved
JACC 2014; 64: 647-656 (SMART-AF) [13]	2014	Nonrandomzied multicenter study of contact force-sensing RF catheter, comparing to performance goals	172	Paroxysmal	PVI, optional CFAEs and lines	12 months	Freedom from symptomatic AF, flutter, tachycardia, acute procedural failure, or changes in AAD	72.5%	N/A	<0.0001	7.5%	NA	FDA approval received
Circulation 2015; 132: 907-915 (TOCCASTAR) [12]	2015	Randomized to contact force sensing RF catheter or approved RF catheter, multicenter	300	Paroxysaml	PVI, optional triggers, CAFEs and lines in both arms	12 months	Acute procedural success + Freedom from Symptomatic AF/Flutter/Tachycardia off AAD	67.8%	69.4%	0.0073 for noninferiority	7.2%	9.1%	FDA approval received
JACC 2015; 66: 1350-1360 (HeartLight) [11]	2015	Randomized to laserballoon or approved RF catheter, multicenter	353	Paroxysmal	PVI ± CTI ablation vs PVI, optional CFAEs, and Lines	12 months	Freedom from Symptomatic AF/Flutter/Tachycardia, acute procedural failure, AAD, or non-prototocol intervention	61.1%	61.7%	0.003 for noninferiority	5.3%	6.4%	FDA approval received
First-Line Therapy Trials													
JAMA 2005; 293: 2634-2640 (RAAFT) [29]	2005	Randomized to drug, multicenter	70	Paroxysmal (*N*=67), persistent (*N*= 3)	PVI	12 months	Freedom from detectable AF	84%	37%	<0.01	9%	11%	
NEJM 2012; 367:1587-1595 (MANTRA-PAF) [30]	2012	Randomized to drug, multicenter	294	Paroxysmal AF	PVI, roof line, optional mitral and tricuspid line	24 months	Cumulative AF burden	13% AF burden	19% AF burden	NS	17%	15%	
JAMA 2014; 311: 692-700 (RAAFT-2) [31]	2014	Randomized to drug multicenter	127	Paroxysmal AF	PVI plus optional non-PVI targets	24 months	Freedom from detectable AF, flutter, tachycardia	45%	28%	0.02	9%	4.9%	
Other Paroxysmal AF Ablation Trials													
JACC 2006; 48: 2340-2347 (APAF) [16]	2006	Randomized to drug single center	198	Paroxysmal AF	PVI, mitral line and tricuspid line	12 months	Freedom from detectable AF, flutter, tachycardia	86%	22%	<0.001	1%	23%	
Circulation 2008; 118: 2498-2505 (A4) [7]	2008	Randomized to drug	112	Paroxysmal	PVI (optional LA lines, CTI, focal)	12 months	Freedom from AF	89%	23%	<0.0001	5.7%	1.7%	
NEJM 2016; 374: 2235-2245 (FIRE AND ICE) [10]	2016	Randomized RF vs Cryo, multicenter	762	Paroxysmal AF	PVI	12 months	Freedom from detectable AF, flutter, tachycardia	64.1% (RF)	65.4% (cryo)	NS	12.8%	10.2%	
JACC 2016; 68: 2747-2757 [15]	2016	Randomized to hot balloon or drug, multicenter	100	Paroxysmal AF	PVI	12 months	Freedom from AF	59%	5%	<0.001	10.4%	4.7%	
Other Persistent AF Ablation Trials													
NEJM 2006; 354: 934-941 [25]	2006	Randomized to RF ablation or to CV and short term amio	146	Persistent	PVI, roof, mitral line	12 months	No AF or flutter month 12	74%	58%	0.05	1.3%	1.4%	
EHJ 2014; 35: 501-507 (SARA) [26]	2014	Randomized to drug (2:1 ablation to drug), multicenter	146	Persistent	PVI (optional LA lines, CFAEs)	12 months	Freedom from AF/flutter lasting >24h	70%	44%	0.002	6.1%	4.20%	
NEJM 2015; 372: 1812-1822 [19]	2015	Randomized ablation strategies, multicenter	589	Persistent	PVI alone versus PVI & CFAEs or PVI & lines	18 months	Freedom from afib with or without drugs	59% (PVI alone)	49% & 46%	NS	6%	4.3% & 7.6%	
Other Mixed Paroxysmal and Persistent AF Ablation Trials													
J Med Assoc Thai 2003; 86 (Suppl 1): S8-S16 [24]	2003	Randomized to RF ablation or amiodarone	30	Paroxysmal (70%), Persistent (30%)	PVI, mitral line, CTI, SVC to IVC	12 months	Freedom from AF	79%	40%	0.018	6.70%	47%	
EHJ 2006; 27: 216-221 [17]	2006	Randomized to RF ablation or drug, multicenter	137	Paroxysmal (67%), Persistent (33%)	PVI, mitral line, CTI	12 months	Freedom from AF, flutter, tachycardia	66%	9%	<0.001	4.40%	2.90%	
JCVEP 2009, 20: 22-28 [18]	2009	Randomized to RF ablation or drug, multicenter	70	Paroxysmal (41%), Persistent (59%) & type 2 DM	PVI, CTI, optional mitral line and roof line	12 months	Freedom from AF and atypical atrial flutter	80%	43%	0.001	2.90%	17%	
Randomized Trials of AF Ablation in Patients with Heart Failure													
NEJM 2008; 359: 1778-1785 (PABA-HF) [38]	2008	Randomized to RF ablation of AVJ abl and BiV pacing	81	Persistent (50%), Paroxysmal (50%), EF 27% abl, 29% AVJ	PVI, optional linear abl and CFAEs	6 months	Composite EF, 6 min walk, MLWHF score; freedom from AF (secondary, mult proc, +/- AA drugs)	88% AF free, EF 35% abl, 28% AVJ (*P* <.001), > QOL and 6 min walk increase with abl		<0.001	14.60%	17.50%	
Heart 2011; 97: 740-747 [39]	2011	Randomized to RF ablation or pharmacological rate control	41	Persistent, EF 20% abl, 16% rate control	PVI, roof line, CFAEs	6 months	Change in LVEF, sinus rhythm at 6 months (secondary)	50% in NSR, LVEF increase 4.5%	0% in NSR, LVEF increase 2.8%	0.6 (for EF increase)	15%	Not reported	
JACC 2013; 61: 1894-1903 [46]	2013	Randomized to RF ablation or pharmacological rate control	52	Persistent AF (100%), EF 22% abl, 25% rate control	PVI, optional linear abl and CFAEs	12 months	Change in peak O_2_ consumption (also reported single procedure off drug ablation success)	Peak O_2_ consumption increase greater with abl, 72% abl success		0.018	15%	Not reported	
Circ A and E 2014; 7: 31-38 [40]	2014	Randomized to RF ablation or pharmacological rate control	50	Persistent AF (100%), EF 32% abl, 34% rate control	PVI, optional linear abl and CFAEs	6 months	Change in LVEF at 6 months, multiple procedure freedom from AF also reported	LVEF 40% with abl, 31% rate control, 81% AF free with abl		0.015	7.70%		

*AF* atrial fibrillation, *RF* radiofrequency, *AVJ* atrioventricular junction, *abl* ablation, *BiV* biventricular, *EF* ejection fraction, *PVI* pulmonary vein isolation, *CFAEs* complex fractionated atrial electrograms, *MLWHF* Minnesota Living with Heart Failure, *LVEF* left ventricular ejection fraction, *QOL* quality of life, *NSR* normal sinus rhythm

## Complications

Catheter ablation of AF is one of the most complex interventional electrophysiological procedures. AF ablation by its nature involves catheter manipulation and ablation in the delicate thin-walled atria, which are in close proximity to other important organs and structures that can be impacted through collateral damage. It is therefore not surprising that AF ablation is associated with a significant risk of complications, some of which might result in life-long disability and/or death. This section reviews the complications associated with catheter ablation procedures performed to treat AF. The types and incidence of complications are presented, their mechanisms are explored, and the optimal approach to prevention and treatment is discussed (Tables 8 and 9).

**Table 8:** Definitions of complications associated with AF ablation

Asymptomatic cerebral embolism	Asymptomatic cerebral embolism is defined as an occlusion of a blood vessel in the brain due to an embolus that does not result in any acute clinical symptoms. Silent cerebral embolism is generally detected using a diffusion weighted MRI.
Atrioesophageal fistula	An atrioesophageal fistula is defined as a connection between the atrium and the lumen of the esophagus. Evidence supporting this diagnosis includes documentation of esophageal erosion combined with evidence of a fistulous connection to the atrium, such as air emboli, an embolic event, or direct observation at the time of surgical repair. A CT scan or MRI scan is the most common method of documentation of an atrioesophageal fistula.
Bleeding	Bleeding is defined as a major complication of AF ablation if it requires and/or is treated with transfusion or results in a 20% or greater fall in hematocrit.
Bleeding following cardiac surgery	Excessive bleeding following a surgical AF ablation procedure is defined as bleeding requiring reoperation or ≥2 units of PRBC transfusion within any 24 h of the first 7 days following the index procedure.
Cardiac perforation	We recommend that cardiac perforation be defined together with cardiac tamponade. See “Cardiac tamponade/perforation.”
Cardiac tamponade	We recommend that cardiac tamponade be defined together with cardiac perforation. See “Cardiac tamponade/perforation.”
Cardiac tamponade/perforation	Cardiac tamponade/perforation is defined as the development of a significant pericardial effusion during or within 30 days of undergoing an AF ablation procedure. A significant pericardial effusion is one that results in hemodynamic compromise, requires elective or urgent pericardiocentesis, or results in a 1-cm or more pericardial effusion as documented by echocardiography. Cardiac tamponade/perforation should also be classified as “early” or “late” depending on whether it is diagnosed during or following initial discharge from the hospital.
Deep sternal wound infection/mediastinitis following cardiac surgery	Deep sternal wound infection/mediastinitis following cardiac surgery requires one of the following: (1) an organism isolated from culture of mediastinal tissue or fluid; (2) evidence of mediastinitis observed during surgery; (3) one of the following conditions: chest pain, sternal instability, or fever (>38°C), in combination with either purulent discharge from the mediastinum or an organism isolated from blood culture or culture of mediastinal drainage.
Esophageal injury	Esophageal injury is defined as an erosion, ulceration, or perforation of the esophagus. The method of screening for esophageal injury should be specified. Esophageal injury can be a mild complication (erosion or ulceration) or a major complication (perforation).
Gastric motility/pyloric spasm disorders	Gastric motility/pyloric spasm disorder should be considered a major complication of AF ablation when it prolongs or requires hospitalization, requires intervention, or results in late disability, such as weight loss, early satiety, diarrhea, or GI disturbance.
Major complication	A major complication is a complication that results in permanent injury or death, requires intervention for treatment, or prolongs or requires hospitalization for more than 48 h. Because early recurrences of AF/AFL/AT are to be expected following AF ablation, recurrent AF/AFL/AT within 3 months that requires or prolongs a patient's hospitalization should not be considered to be a major complication of AF ablation.
Mediastinitis	Mediastinitis is defined as inflammation of the mediastinum. Diagnosis requires one of the following: (1) an organism isolated from culture of mediastinal tissue or fluid; (2) evidence of mediastinitis observed during surgery; (3) one of the following conditions: chest pain, sternal instability, or fever (>38°C), in combination with either purulent discharge from the mediastinum or an organism isolated from blood culture or culture of mediastinal drainage.
Myocardial infarction in the context of AF ablation	The universal definition of myocardial infarction [395] cannot be applied in the context of catheter or surgical AF ablation procedures because it relies heavily on cardiac biomarkers (troponin and CPK), which are anticipated to increase in all patients who undergo AF ablation as a result of the ablation of myocardial tissue. Similarly, chest pain and other cardiac symptoms are difficult to interpret in the context of AF ablation both because of the required sedation and anesthesia and also because most patients experience chest pain following the procedure as a result of the associated pericarditis that occurs following catheter ablation. We therefore propose that a myocardial infarction, in the context of catheter or surgical ablation, be defined as the presence of any one of the following criteria: (1) detection of ECG changes indicative of new ischemia (new ST-T wave changes or new LBBB) that persist for more than 1 h; (2) development of new pathological Q waves on an ECG; (3) imaging evidence of new loss of viable myocardium or new regional wall motion abnormality.
Pericarditis	Pericarditis should be considered a major complication following ablation if it results in an effusion that leads to hemodynamic compromise or requires pericardiocentesis, prolongs hospitalization by more than 48 h, requires hospitalization, or persists for more than 30 days following the ablation procedure.
Phrenic nerve paralysis	Phrenic nerve paralysis is defined as absent phrenic nerve function as assessed by a sniff test. A phrenic nerve paralysis is considered to be permanent when it is documented to be present 12 months or longer following ablation.
Pulmonary vein stenosis	Pulmonary vein stenosis is defined as a reduction of the diameter of a PV or PV branch. PV stenosis can be categorized as mild <50%, moderate 50%–70%, and severe ≥70% reduction in the diameter of the PV or PV branch. A severe PV stenosis should be considered a major complication of AF ablation.
Serious adverse device effect	A serious adverse device effect is defined as a serious adverse event that is attributed to use of a particular device.
Stiff left atrial syndrome	Stiff left atrial syndrome is a clinical syndrome defined by the presence of signs of right heart failure in the presence of preserved LV function, pulmonary hypertension (mean PA pressure >25 mmHg or during exercise >30 mmHg), and large V waves ≥10 mmHg or higher) on PCWP or left atrial pressure tracings in the absence of significant mitral valve disease or PV stenosis.
Stroke or TIA postablation	Stroke diagnostic criteria
	•Rapid onset of a focal or global neurological deficit with at least one of the following: change in level of consciousness, hemiplegia, hemiparesis, numbness or sensory loss affecting one side of the body, dysphasia or aphasia, hemianopia, amaurosis fugax, or other neurological signs or symptoms consistent with stroke
	•Duration of a focal or global neurological deficit ≥24 h; OR <24 h if therapeutic intervention(s) were performed (e.g., thrombolytic therapy or intracranial angioplasty); OR available neuroimaging documents a new hemorrhage or infarct; OR the neurological deficit results in death.
	•No other readily identifiable nonstroke cause for the clinical presentation (e.g., brain tumor, trauma, infection, hypoglycemia, peripheral lesion, pharmacological influences).^∗^
	•Confirmation of the diagnosis by at least one of the following: neurology or neurosurgical specialist; neuroimaging procedure (MRI or CT scan or cerebral angiography); lumbar puncture (i.e., spinal fluid analysis diagnostic of intracranial hemorrhage)
	Stroke definitions
	• Transient ischemic attack: new focal neurological deficit with rapid symptom resolution (usually 1 to 2 h), always within 24 h; neuroimaging without tissue injury
	•Stroke: (diagnosis as above, preferably with positive neuroimaging study);
	Minor—Modified Rankin score <2 at 30 and 90 days^†^
	Major—Modified Rankin score ≥2 at 30 and 90 days
Unanticipated adverse device effect	Unanticipated adverse device effect is defined as complication of an ablation procedure that has not been previously known to be associated with catheter or surgical ablation procedures.
Vagal nerve injury	Vagal nerve injury is defined as injury to the vagal nerve that results in esophageal dysmotility or gastroparesis. Vagal nerve injury is considered to be a major complication if it prolongs hospitalization, requires hospitalization, or results in ongoing symptoms for more than 30 days following an ablation procedure.
Vascular access complication	Vascular access complications include development of a hematoma, an AV fistula, or a pseudoaneurysm. A major vascular complication is defined as one that requires intervention, such as surgical repair or transfusion, prolongs the hospital stay, or requires hospital admission.

*AF* atrial fibrillation, *CT* computed tomography, *MRI* magnetic resonance imaging, *PRBC* packed red blood cell, *AFL* atrial flutter, *AT* atrial tachycardia, *CPK* creatine phosphokinase, *ECG* electrocardiogram, *LBBB* left bundle branch block

^∗^Patients with nonfocal global encephalopathy will not be reported as a stroke without unequivocal evidence based on neuroimaging studies

^†^Modified Rankin score assessments should be made by qualified individuals according to a certification process. If there is discordance between the 30- and 90-day modified Rankin scores, a final determination of major versus minor stroke will be adjudicated by the neurology members of the clinical events committee

**Table 9:** Incidence, prevention, diagnosis, and treatment of selected complications of AF ablation

Complication	Incidence	Selected prevention techniques	Diagnostic testing	Selected treatment options	References
Air embolism	<1%	Sheath management	Nothing or cardiac catheterization	Supportive care with fluid, oxygen, head down tilt, hyperbaric oxygen	[388, 396–401]
Asymptomatic cerebral emboli (ACE)	2% to 15%	Anticoagulation, catheter and sheath management, TEE	Brain MRI	None	[402–419]
Atrial esophageal fistula	0.02% to 0.11%	Reduce power, force, and RF time on posterior wall, monitor esophageal temp, use proton pump inhibitors; avoid energy delivery over esophagus	CT scan of chest, MRI; avoid endoscopy with air insufflation	Surgical repair	[337–365, 420–456]
Cardiac tamponade	0.2% to 5%	Cather manipulation, transseptal technique, reduce power, force, and RF time	Echocardiography	Pericardiocentesis or surgical drainage	[338, 343, 347, 457–467]
Coronary artery stenosis/occlusion	<0.1%	Avoid high-power energy delivery near coronary arteries	Cardiac catheterization	PTCA	[468–476]
Death	<0.1% to 0.4%	Meticulous performance of procedure, attentive postprocedure care	NA	NA	[338, 343, 347, 458, 477]
Gastric hypomotility	0% to 17%	Reduce power, force, and RF time on posterior wall	Endoscopy, barium swallow, gastric emptying study	Metoclopramide, possibly intravenous erythromycin	[478–490]
Mitral valve entrapment	<0.1%	Avoid circular catheter placement near or across mitral valve; clockwise torque on catheter	Echocardiography	Gentle catheter manipulation, surgical extraction	[491–498]
Pericarditis	0% to 50%	None proven	Clinical history, ECG, sedimentation rate, echocardiogram	NSAID, colchicine, steroids	[499–506]
Permanent phrenic nerve paralysis	0% to 0.4%	Monitor diaphragm during phrenic pacing, CMAP monitoring, phrenic pacing to identify location and adjust lesion location	CXR, sniff test	Supportive care	[9, 11, 156, 347, 367, 446, 457, 478, 479, 487–490, 507–528]
Pulmonary vein stenosis	<1%	Avoid energy delivery within PV	CT or MRI, V/Q wave scan	Angioplasty, stent, surgery	[9, 11, 313, 316–335, 457, 529–531]
Radiation injury	<0.1%	Minimize fluoroscopy exposure, especially in obese and repeat ablation patients, X-ray equipment	None	Supportive care, rarely skin graft	[513, 532–550]
Stiff left atrial syndrome	<1.5%	Limit extent of left atrial ablation	Echocardiography, cardiac catheterization	Diuretics	[551–558]
Stroke and TIA	0% to 2%	Pre-, post-, and intraprocedure anticoagulation, catheter and sheath management, TEE	Head CT or MRI, cerebral angiography	Thrombolytic therapy, angioplasty	[10–13, 338, 347, 367, 458, 559–565]
Vascular complications	0.2% to 1.5%	Vascular access techniques, ultrasound-guided access, anticoagulation management	Vascular ultrasound, CT scan	Conservative treatment, surgical repair, transfusion	[338, 347, 371, 373, 374, 380, 458, 511, 566–575]

*AF* atrial fibrillation, *CT* computed tomography, *MRI* magnetic resonance imaging, *TEE* transesophageal electrocardiogram, *RF* radiofrequency, *PTCA* percutaneous transluminal coronary angioplasty, *NA* not applicable, *ECG* electrocardiogram, *NSAID* nonsteroidal anti-inflammatory drug, *CMAP* compound motor action potentials, *CXR* chest X-ray, *TIA* transient ischemic attack

## Training requirements

This section of the document outlines the training requirements for those who wish to perform catheter ablation of AF.

## Surgical and hybrid AF ablation

Please refer to Table 2 and Fig. 8 presented earlier in this Executive Summary.

## Clinical trial design

Although there have been many advances made in the field of catheter and surgical ablation of AF, there is still much to be learned about the mechanisms of initiation and maintenance of AF and how to apply this knowledge to the still-evolving techniques of AF ablation. Although single-center, observational reports have dominated the early days of this field, we are quickly moving into an era in which hypotheses are put through the rigor of testing in well-designed, randomized, multicenter clinical trials. It is as a result of these trials that conventional thinking about the best techniques, success rates, complication rates, and long-term outcomes beyond AF recurrence—such as thromboembolism and mortality—is being put to the test. The ablation literature has also seen a proliferation of meta-analyses and other aggregate analyses, which reinforce the need for consistency in the approach to reporting the results of clinical trials. This section reviews the minimum requirements for reporting on AF ablation trials. It also acknowledges the potential limitations of using specific primary outcomes and emphasizes the need for broad and consistent reporting of secondary outcomes to assist the end-user in determining not only the scientific, but also the clinical relevance of the results (Tables 10, 11, 12, and 13).

**Table 10:** Definitions for use when reporting outcomes of AF ablation and in designing clinical trials of catheter or surgical ablation of AF

Acute procedural success (pulmonary vein isolation)	Acute procedural success is defined as electrical isolation of all pulmonary veins. A minimal assessment of electrical isolation of the PVs should consist of an assessment of entrance block. If other methods are used to assess PVI, including exit block and/or the use of provocative agents such as adenosine or isoproterenol, they should be prespecified. Furthermore, it is recommended that the wait time used to screen for early recurrence of PV conduction once initial electrical isolation is documented be specified in all prospective clinical trials.
Acute procedural success (not related by pulmonary vein isolation)	Typically, this would apply to substrate ablation performed in addition to PVI for persistent AF. Although some have proposed AF termination as a surrogate for acute procedural success, its relationship to long-term success is controversial. Complete elimination of the additional substrate (localized rotational activation, scar region, non-PV trigger, or other target) and/or demonstration of bidirectional conduction block across a linear ablation lesion would typically be considered the appropriate endpoint.
One-year success^∗^	One-year success is defined as freedom from AF/AFL/AT after removal from antiarrhythmic drug therapy as assessed from the end of the 3month blanking period to 12 months following the ablation procedure. Because cavotricuspid isthmus-dependent atrial flutter is easily treated with cavotricuspid isthmus ablation and is not an iatrogenic arrhythmia following a left atrial ablation procedure for AF, it is reasonable for clinical trials to choose to prespecify that occurrence of isthmus-dependent atrial flutter, if confirmed by entrainment maneuvers during electrophysiology testing, should not be considered an ablation failure or primary effectiveness endpoint.
Alternative one-year success	Although the one-year success definition provided above remains the recommended end point that should be reported in all AF ablation trials, and the endpoint for which the objective performance criteria listed below were developed, the Task Force recognizes that alternative definitions for success can be used if the main goal of therapy in the study is to relieve AF-related symptoms and to improve patient QOL. In particular, it is appropriate for clinical trials to define success as freedom from only symptomatic AF/AFL/AT after removal from antiarrhythmic drug therapy as assessed from the end of the 3-month blanking period to 12 months following the ablation procedure if the main goal of therapy in the study is to relieve AF-related symptoms and to improve patient QOL. However, because symptoms of AF can resolve over time, and because studies have shown that asymptomatic AF represents a greater proportion of all AF postablation than prior to ablation, clinical trials need to continue to report freedom from both symptomatic and asymptomatic AF even if this alternative one year success definition is used as the primary trial endpoint.
Clinical/partial success^∗^	It is reasonable for clinical trials to define and incorporate one or more secondary definitions of success that can be referred to as “clinical success” or “partial success.” If these alternative definitions of success are included, they should be defined prospectively. In prior Consensus Documents the Task Force has proposed that clinical/partial success be defined as a “75% or greater reduction in the number of AF episodes, the duration of AF episodes, or the % time a patient is in AF as assessed with a device capable of measuring AF burden in the presence or absence of previously ineffective antiarrhythmic drug therapy.” Because there is no firm scientific basis for selecting the cutoff of 75% rather than a different cutoff, this prior recommendation is provided only as an example of what future clinical trials may choose to use as a definition of clinical/partial success.
Long-term success^∗^	Long-term success is defined as freedom from AF/AFL/AT recurrences following the 3-month blanking period through a minimum of 36-month follow-up from the date of the ablation procedure in the absence of Class I and III antiarrhythmic drug therapy.
Recurrent AF/AFL/AT	Recurrent AF/AFL/AT is defined as AF/AFL/AT of at least 30 s' duration that is documented by an ECG or device recording system and occurs following catheter ablation. Recurrent AF/AFL/AT may occur within or following the post ablation blanking period. Recurrent AF/AFL/AT that occurs within the postablation blanking period is not considered a failure of AF ablation.
Early recurrence of AF/AFL/AT	Early recurrence of AF/AFL/AT is defined as a recurrence of atrial fibrillation within three months of ablation. Episodes of atrial tachycardia or atrial flutter should also be classified as a “recurrence.” These are not counted toward the success rate if a blanking period is specified.
Recurrence of AF/AFL/AT	Recurrence of AF/AFL/AT postablation is defined as a recurrence of atrial fibrillation more than 3 months following AF ablation. Episodes of atrial tachycardia or atrial flutter should also be classified as a “recurrence.”
Late recurrence of AF/AFL/AT	Late recurrence of AF/AFL/AT is defined as a recurrence of atrial fibrillation 12 months or more after AF ablation. Episodes of atrial tachycardia or atrial flutter should also be classified as a “recurrence.”
Blanking period	A blanking period of three months should be employed after ablation when reporting efficacy outcomes. Thus, early recurrences of AF/AFL/AT within the first 3 months should not be classified as treatment failure. If a blanking period of less than 3 months is chosen, it should be prespecified and included in the Methods section.
Stroke screening	A risk-based approach to determine the level of postablation stroke screening in clinical trials is recommended by the Task Force. For ablation devices with a lower risk of stroke and for which a stroke signal has not been reported, a minimum standardized neurological assessment of stroke should be conducted by a physician at baseline and at hospital discharge or 24 h after the procedure, whichever is later. If this neurological assessment demonstrates new abnormal findings, the patient should have a formal neurological consult and examination with appropriate imaging (i.e., DW-MRI), used to confirm any suspected diagnosis of stroke. For devices in which a higher risk of stroke is suspected or revealed in prior trials, a formal neurological examination by a neurologist at discharge or 24 h after the procedure, whichever is later, is recommended. Appropriate imaging should be obtained if this evaluation reveals a new neurological finding. In some studies in which delayed stroke is a concern, repeat neurological screening at 30 days postablation might be appropriate.
Detectable AF/AFL/AT	Detectable AF is defined as AF/AFL/AT of at least 30 s' duration when assessed with ECG monitoring. If other monitoring systems are used, including implantable pacemakers, implantable defibrillators, and subcutaneous ECG monitoring devices, the definition of detectable AF needs to be prespecified in the clinical trial based on the sensitivity and specificity of AF detection with the particular device. We recommend that episodes of atrial flutter and atrial tachycardia be included within the broader definition of a detectable AF/AFL/AT episode.
AF/AFL/AT burden	It is reasonable for clinical trials to incorporate AF/AFL/AT burden as a secondary endpoint in a clinical trial of AF ablation. In stating this it is recognized that there are no conclusive data that have validated a rate of AF burden reduction as a predictor of patient benefit (i.e. reduction in mortality and major morbidities such as stroke, CHF, QOL, or hospitalization). If AF burden is included, it is important to predefine and standardize the monitoring technique that will be used to measure AF burden. Available monitoring techniques have been discussed in this document. Should AF burden be selected as an endpoint in a clinical trial, the chosen monitoring technique should be employed at least a month prior to ablation to establish a baseline burden of AF.
Entrance block	Entrance block is defined as the absence, or if present, the dissociation, of electrical activity within the PV antrum. Entrance block is most commonly evaluated using a circular multielectrode mapping catheter positioned at the PV antrum. Entrance block can also be assessed using detailed point-by-point mapping of the PV antrum guided by an electroanatomical mapping system. The particular method used to assess entrance block should be specified in all clinical trials. Entrance block of the left PVs should be assessed during distal coronary sinus or left atrial appendage pacing in order to distinguish far-field atrial potentials from PV potentials. It is recommended that reassessment of entrance block be performed a minimum of 20 min after initial establishment of PV isolation.
Procedural endpoints for AF ablation strategies not targeting the PVs	Procedural endpoints for AF ablation strategies not targeting the PVs: The acute procedural endpoints for ablation strategies not targeting the PVs vary depending on the specific ablation strategy and tool. It is important that they be prespecified in all clinical trials. For example, if a linear ablation strategy is used, documentation of bidirectional block across the ablation line must be shown. For ablation of CFAEs, rotational activity, or non-PV triggers, the acute endpoint should at a minimum be elimination of CFAEs, rotational activity, or non-PV triggers. Demonstration of AF slowing or termination is an appropriate procedural endpoint, but it is not required as a procedural endpoint for AF ablation strategies not targeting the PVs.
Esophageal temperature monitoring	Esophageal temperature monitoring should be performed in all clinical trials of AF ablation. At a minimum, a single thermocouple should be used. The location of the probe should be adjusted during the procedure to reflect the location of energy delivery. Although this document does not provide formal recommendations regarding the specific temperature or temperature change at which energy delivery should be terminated, the Task Force does recommend that all trials prespecify temperature guidelines for termination of energy delivery.
Enrolled subject	An enrolled subject is defined as a subject who has signed written informed consent to participate in the trial in question.
Exit block	Exit block is defined as the inability to capture the atrium during pacing at multiple sites within the PV antrum. Local capture of musculature within the pulmonary veins and/or antrum must be documented to be present to make this assessment. Exit block is demonstrated by a dissociated spontaneous pulmonary vein rhythm.
Nonablative strategies	The optimal nonablative therapy for patients with persistent and long-standing persistent AF who are randomized to the control arm of an AF ablation trial is a trial of a new Class I or III antiarrhythmic agent or a higher dose of a previously failed antiarrhythmic agent. For patients with persistent or long-standing persistent AF, performance of a direct-current cardioversion while taking the new or dose adjusted antiarrhythmic agent should be performed, if restoration of sinus rhythm is not achieved following initiation and/or dose adjustment of antiarrhythmic drug therapy. Failure of pharmacological cardioversion alone is not adequate to declare this pharmacological strategy unsuccessful.
Noninducibility of atrial fibrillation	Noninducibility of atrial fibrillation is defined as the inability to induce atrial fibrillation with a standardized prespecified pharmacological or electrical stimulation protocol. The stimulation protocol should be prespecified in the specific clinical trial. Common stimulation approaches include a high-dose isoproterenol infusion protocol or repeated atrial burst pacing at progressively more rapid rates.
Patient populations for inclusion in clinical trials	It is considered optimal for clinical trials to enroll patients with only one type of AF: paroxysmal, persistent, or long-standing persistent. If more than one type of AF patient is enrolled, the results of the trial should also be reported separately for each of the AF types. It is recognized that “early persistent” AF responds to AF ablation to a similar degree as patients with paroxysmal AF and that the response of patients with “late persistent AF” is more similar to that in those with long-standing persistent AF.
Therapy consolidation period	Following a 3-month blanking period, it is reasonable for clinical trials to incorporate an additional 1- to 3-month therapy consolidation period. During this time, adjustment of antiarrhythmic medications and/or cardioversion can be performed. Should a consolidation period be incorporated into a clinical trial design, the minimum follow-up duration should be 9 months following the therapy consolidation period. Performance of a repeat ablation procedure during the blanking or therapy consolidation period would “reset” the endpoint of the study and trigger a new 3-month blanking period. Incorporation of a therapy consolidation period can be especially appropriate for clinical trials evaluating the efficacy of AF ablation for persistent or long-standing persistent AF. The challenge of this approach is that it prolongs the overall study duration. Because of this concern regarding overall study duration, we suggest that the therapy consolidation period be no more than 3 months in duration following the 3-month blanking period.
Recommendations regarding repeat ablation procedures	It is recommended that all clinical trials report the single procedure efficacy of catheter ablation. Success is defined as freedom from symptomatic or asymptomatic AF/AFL/AT of 30 s or longer at 12 months postablation. Recurrences of AF/AFL/AT during the first 3-month blanking period post-AF ablation are not considered a failure. Performance of a repeat ablation procedure at any point after the initial ablation procedure should be considered a failure of a single procedure strategy. It is acceptable for a clinical trial to choose to prespecify and use a multiprocedure success rate as the primary endpoint of a clinical trial. When a multiprocedure success is selected as the primary endpoint, efficacy should be defined as freedom from AF/flutter or tachycardia at 12 months after the final ablation procedure. In the case of multiple procedures, repeat ablation procedures can be performed at any time following the initial ablation procedure. All ablation procedures are subject to a 3-month post blanking window, and all ablation trials should report efficacy at 12 months after the final ablation procedure.
Cardioversion definitions	
Failed electrical cardioversion	Failed electrical cardioversion is defined as the inability to restore sinus rhythm for 30 s or longer following electrical cardioversion.
Successful electrical cardioversion	Successful electrical cardioversion is defined as the ability to restore sinus rhythm for at least 30 s following cardioversion.
Immediate AF recurrence postcardioversion	Immediate AF recurrence postcardioversion is defined as a recurrence of AF within 24 h following cardioversion. The most common time for an immediate recurrence is within 30–60 min postcardioversion.
Early AF recurrence postcardioversion	Early AF recurrence postcardioversion is defined as a recurrence of AF within 30 days of a successful cardioversion.
Late AF recurrence postcardioversion	Late AF recurrence postcardioversion is defined as recurrence of AF more than 30 days following a successful cardioversion.
Surgical ablation definitions	
Hybrid AF surgical ablation procedure	Hybrid AF surgical ablation procedure is defined as a joint AF ablation procedure performed by electrophysiologists and cardiac surgeons either as part of a single “joint” procedure or performed as two preplanned separate ablation procedures separated by no more than 6 months.
Surgical Maze ablation procedure	Surgical Maze ablation procedure is defined as a surgical ablation procedure for AF that includes, at a minimum, the following components: (1) line from SVC to IVC; (2) line from IVC to the tricuspid valve; (3) isolation of the PVs; (4) isolation of the posterior left atrium; (5) line from MV to the PVs; (6) management of the LA appendage.
Stand-alone surgical AF ablation	A surgical AF ablation procedure during which other cardiac surgical procedures are not performed such as CABG, valve replacement, or valve repair.
Nomenclature for types of surgical AF ablation procedures	We recommend that the term “Maze” procedure is appropriately used only to refer to the biatrial lesion set of the Cox-Maze operation. It requires ablation of the RA and LA isthmuses. Less extensive lesion sets should not be referred to as a “Maze” procedure, but rather as a surgical AF ablation procedure. In general, surgical ablation procedures for AF can be grouped into three different groups: (1) a full biatrial Cox-Maze procedure; (2) PVI alone; and (3) PVI combined with left atrial lesion sets.
Hybrid epicardial and endocardial AF ablation	This term refers to a combined AF ablation procedure involving an off-pump minimally invasive surgical AF ablation as well as a catheter-based AF ablation procedure designed to complement the surgical lesion set. Hybrid ablation procedures may be performed in a single-procedure setting in a hybrid operating room or a cardiac catheterization laboratory environment, or it can be staged. When staged, it is most typical to have the patient undergo the minimally invasive surgical ablation procedure first following by a catheter ablation procedure 1 to 3 months later. This latter approach is referred to as a “staged Hybrid AF ablation procedure.”
Minimum AF documentation, endpoints, TEE performance, and success rates in clinical trials	
Minimum documentation for paroxysmal AF	The minimum AF documentation requirement for paroxysmal AF is (1) physician's note indicating recurrent self-terminating AF and (2) one electrocardiographically documented AF episode within 6 months prior to the ablation procedure.
Minimum documentation for persistent AF	The minimum AF documentation requirement for persistent AF is (1) physician's note indicating continuous AF >7 days but no more than 1 year and (2) a 24-h Holter within 90 days of the ablation procedure showing continuous AF.
Minimum documentation for early persistent AF	The minimum AF documentation requirement for persistent AF is (1) physician's note indicating continuous AF >7 days but no more than 3 months and (2) a 24-h Holter showing continuous AF within 90 days of the ablation procedure.
Minimum documentation for long-standing persistent AF	The minimum AF documentation requirement for long-standing persistent AF is as follows: physician's note indicating at least 1 year of continuous AF plus a 24-h Holter within 90 days of the ablation procedure showing continuous AF. The performance of a successful cardioversion (sinus rhythm >30 s) within 12 months of an ablation procedure with documented early recurrence of AF within 30 days should not alter the classification of AF as long-standing persistent.
Symptomatic AF/AFL/AT	AF/AFL/AT that results in symptoms that are experienced by the patient. These symptoms can include but are not limited to palpitations, presyncope, syncope, fatigue, and shortness of breath. For patients in continuous AF, reassessment of symptoms after restoration of sinus rhythm is recommended to establish the relationship between symptoms and AF.
Documentation of AF-related symptoms	Documentation by a physician evaluating the patient that the patient experiences symptoms that could be attributable to AF. This does not require a time-stamped ECG, Holter, or event monitor at the precise time of symptoms. For patients with persistent AF who initially report no symptoms, it is reasonable to reassess symptom status after restoration of sinus rhythm with cardioversion.
Minimum effectiveness endpoint for patients with symptomatic and asymptomatic AF	The minimum effectiveness endpoint is freedom from symptomatic and asymptomatic episodes of AF/AFL/AT recurrences at 12 months following ablation, free from antiarrhythmic drug therapy, and including a prespecified blanking period.
Minimum chronic acceptable success rate: paroxysmal AF at 12-month follow-up	If a minimum chronic success rate is selected as an objective effectiveness endpoint for a clinical trial, we recommend that the minimum chronic acceptable success rate for paroxysmal AF at 12-month follow-up is 50%.
Minimum chronic acceptable success rate: persistent AF at 12-month follow-up	If a minimum chronic success rate is selected as an objective effectiveness endpoint for a clinical trial, we recommend that the minimum chronic acceptable success rate for persistent AF at 12-month follow-up is 40%.
Minimum chronic acceptable success rate: long-standing persistent AF at 12-month follow-up	If a minimum chronic success rate is selected as an objective effectiveness endpoint for a clinical trial, we recommend that the minimum chronic acceptable success rate for long-standing persistent AF at 12-month follow-up is 30%.
Minimum follow-up screening for paroxysmal AF recurrence	For paroxysmal AF, the minimum follow-up screening should include (1) 12-lead ECG at each follow-up visit; (2) 24-h Holter at the end of the follow-up period (e.g., 12 months); and (3) event recording with an event monitor regularly and when symptoms occur from the end of the 3-month blanking period to the end of follow-up (e.g., 12 months).
Minimum follow-up screening for persistent or long-standing AF recurrence	For persistent and long-standing persistent AF, the minimum follow-up screening should include (1) 12-lead ECG at each follow-up visit; (2) 24-h Holter every 6 months; and (3) symptom-driven event monitoring.
Requirements for transesophageal echocardiogram	It is recommended that the minimum requirement for performance of a TEE in a clinical trial should be those requirements set forth in ACC/AHA/HRS 2014 Guidelines for AF Management pertaining to anticoagulation at the time of cardioversion. Prior to undergoing an AF ablation procedure a TEE should be performed in all patients with AF of >48 h' duration or of unknown duration if adequate systemic anticoagulation has not been maintained for at least 3 weeks prior to AF ablation. If a TEE is performed for this indication, it should be performed within 24 h of the ablation procedure.

*AF* atrial fibrillation, *DW-MRI* diffusion-weighted magnetic resonance imaging, *CHF* congestive heart failure, *QOL* quality of life, *ECG* electrocardiogram, *CABG* coronary artery bypass grafting, *PV* pulmonary vein, *SVC* superior vena cava, *IVC* inferior vena cava, *CFAE* complex fractionated atrial electrogram, *PVI* pulmonary vein isolation, *AFL* atrial flutter, *AT* atrial tachycardia, *ACC* American College of Cardiology, *AHA* American Heart Association, *HRS* Heart Rhythm Society

^∗^When reporting outcomes of AF ablation, the development of atrial tachycardia or atrial flutter should be included in the broad definition of recurrence following AF ablation. All studies should report freedom from AF, atrial tachycardia, and atrial flutter. These endpoints can also be reported separately. All studies should also clearly specify the type and frequency of ECG monitoring as well as the degree of compliance with the prespecified monitoring protocol

**Table 11:** Quality-of-life scales, definitions, and strengths

Scale	Definition/Details	Strengths/Weaknesses
Short Form (36) Health Survey (SF36)38(General)	Consists of 8 equally weighted, scaled scores in the following sections: vitality, physical functioning, bodily pain, general health perceptions, physical role functioning, emotional role functioning, social role functioning, mental health. Each section receives a scale score from 0 to 100.Physical component summary (PCS) and mental component summary (MCS) is an average of all the physically and mentally relevant questions, respectively.The Short Form (12) Health Survey (SF12) is a shorter version of the SF-36, which uses just 12 questions and still provides scores that can be compared with SF-36 norms, especially for summary physical and mental functioning.Gives more precision in measuring QOL than EQ-5D but can be harder to transform into cost utility analysis.	Advantages: extensively validated in a number of disease and health states. Might have more resolution than EQ-50 for AF QOL.Disadvantages: not specific for AF, so might not have resolution to detect AF-specific changes in QOL.
EuroQol Five Dimensions Questionnaire (EQ-5D)39(General)	Two components: Health state description is measured in five dimensions: mobility, self-care, usual activities, pain/discomfort, anxiety/depression. Answers may be provided on a three-level (3L) or five-level (5L) scale. In the Evaluation section, respondents evaluate their overall health status using a visual analogue scale (EQ-VAS). Results can easily be converted to quality-adjusted life years for cost utility analysis.	Advantages: extensively validated in a number of disease and health states. Can easily be converted into quality-adjusted life years for cost-effectiveness analysis.Disadvantages: might not be specific enough to detect AF-specific changes in QOL. Might be less specific than SF-36.
AF effect on Quality of Life Survey (AFEQT)40 (AF specific)	20 questions: 4 targeting AF-related symptoms, 8 evaluating daily function, and 6 assessing AF treatment concerns. Each item scored on a 7-point Likert scale.	Advantages: brief, simple, very responsive to AF interventions. Good internal validity and well validated against a number of other global and AF-specific QOL scales. Used in CABANA.Disadvantages: validation in only two published studies (approximately 219 patients).
Quality of Life Questionnaire for Patients with AF(AF-QoL)41(AF specific)	18-item self-administered questionnaire with three domains: psychological, physical, and sexual activity. Each item scores on a 5-point Likert scale.	Advantages: brief, simple, responsive to AF interventions; good internal validity; used in SARA trial.Disadvantages: external validity compared only to SF-36; formal validation in 1 study (approximately 400 patients).
Arrhythmia-Related Symptom Checklist (SCL)42 (AF specific)	16 items covering AF symptom frequency and symptom severity.	Advantages: most extensively validated in a number of arrhythmia cohorts and clinical trials.Disadvantages: time-consuming and uncertain generalizability.
Mayo AF Specific Symptom Inventory (MAFSI)43 (AF specific)	10 items covering AF symptom frequency and severity. Combination of 5- point and 3-point Likert scale responses.Used in CABANA trial.	Advantages: validated in an AF ablation population and responsive to ablation outcome; used in CABANA trial.Disadvantages: external validity compared only to SF-36; 1 validation study (approximately 300 patients).
University of Toronto Atrial Fibrillation Severity Scale (AFSS) (AF specific)44	10 items covering frequency, duration, and severity. 7-point Likert scale responses.	Advantages: validated and reproducible; used in CTAF trial.Disadvantages: time-consuming and uncertain generalizability.
Arrhythmia Specific Questionnaire in Tachycardia and Arrhythmia (ASTA)45 (AF specific)	Records number of AF episodes and average episode duration during last 3 months. 8 symptoms and 2 disabling symptoms are recorded with scores from 1–4 for each.	Advantages: validated in various arrhythmia groups; external validity compared with SCL, EQ5D, and SF-36; used in MANTRA-PAF; brief; simple.Disadvantages: one validation study (approximately 300 patients).
European Heart Rhythm Association (EHRA)46 (AF specific)	Like NYHA scale. I = no symptoms, II = mild symptoms not affecting daily activity, III = severe symptoms affecting daily activity, and IV = disabling symptoms terminating daily activities.	Advantage: very simple, like NYHA.Disadvantages: not used in studies and not well validated; not very specific; unknown generalizability.
Canadian Cardiovascular Society Severity of Atrial Fibrillation Scale (CCS-SAF)47 (AF specific)	Like NYHA scale. O = asymptomatic, I = AF symptoms have minimal effect on patient's QOL, II = AF symptoms have minor effect on patient QOL, III = symptoms have moderate effect on patient QOL, IV= AF symptoms have severe effect on patient QOL.	Advantages: very simple, like NYHA; validated against SF-36 and University of Toronto AFSS.Disadvantages: poor correlation with subjectiveAF burden; not very specific.

*AF* atrial fibrillation, *QOL* quality of life, *CABANA* Catheter Ablation vs Anti-arrhythmic Drug Therapy for Atrial Fibrillation, *SARA* Study of Ablation Versus antiaRrhythmic Drugs in Persistent Atrial Fibrillation, *CTAF* Canadian Trial of Atrial Fibrillation, *MANTRA-PAF* Medical ANtiarrhythmic Treatment or Radiofrequency Ablation in Paroxysmal Atrial Fibrillation, *NYHA* New York Heart Association, *AFSS* atrial fibrillation severity scale

**Table 12:** Non-AF recurrence–related endpoints for reporting in AF ablation trials

Stroke and bleeding endpoints	Definitions/Details
Stroke (2014 ACC/AHA Key Data Elements)	An acute episode of focal or global neurological dysfunction caused by brain, spinal cord, or retinal vascular injury as a result of hemorrhage or infarction. Symptoms or signs must persist ≥24 h, or if documented by CT, MRI or autopsy, the duration of symptoms/signs may be less than 24 h. Stroke may be classified as ischemic (including hemorrhagic transformation of ischemic stroke), hemorrhagic, or undetermined. Stroke disability measurement is typically performed using the modified Rankin Scale (mRS).
Transient ischemic attack (2014 ACC/AHA Key Data Elements)	Transient episode of focal neurological dysfunction caused by brain, spinal cord, or retinal ischemia without acute infarction and with signs and symptoms lasting less than 24 h.
Major bleeding (ISTH definition)	Fatal bleeding AND/OR symptomatic bleeding in a critical area or organ, such as intracranial, intraspinal, intraocular, retroperitoneal, intraarticular, pericardial, or intramuscular with compartment syndrome AND/OR bleeding causing a fall in hemoglobin level of 2 g/dL (1.24 mmol/L) or more, or leading to transfusion of two or more units of blood.
Clinically relevant nonmajor bleed (ISTH definition)	An acute or subacute clinically overt bleed that does not meet the criteria for a major bleed but prompts a clinical response such that it leads to one of the following: hospital admission for bleeding; physician-guided medical or surgical treatment for bleeding; change in antithrombotic therapy (including interruption or discontinuation).
Minor bleeding (ISTH definition)	All nonmajor bleeds. Minor bleeds are further divided into clinically relevant and not.
Incidence and discontinuation of oral anticoagulation	The number of patients receiving oral anticoagulation and the type of oral anticoagulation should be documented at the end of follow-up. If patients have their oral anticoagulation discontinued, the number of patients discontinuing, the timing of discontinuation, and the reasons for discontinuation of oral anticoagulation, as well as the clinical characteristics and stroke risk profile of the patients should be reported.

*AF* atrial fibrillation, *CT* computed tomography, *MRI* magnetic resonance imaging

**Table 13:** Advantages and disadvantages of AF-related endpoints in AF ablation trials

Endpoint	Advantages	Disadvantages	Relevance and Comments
Freedom from AF/AFL/AT recurrence “gold standard” is 30 s	- Has been in use for many years	- Can systematically underestimate the efficacy of AF ablation, particularly for persistent AF, if 30-s cutoff is used	- Particularly well suited for paroxysmal AF outcomes
	- Can be used to compare results of new trials with historical trials		- Reporting of cutoffs other than 30 s encouraged as secondary endpoints to better contextualize results
	- Sets a high bar for AF elimination		- May be reported as proportion of patients free from arrhythmia or time to recurrence
Freedom from stroke-relevant AF/AFL/AT-duration cutoff of 1 h	- Useful for trials in which interest is more for prognostic change conferred by ablation rather than elimination of all arrhythmias	- No consistent definition of what a stroke-relevant duration of AF is: ranges from 6 min to 24 h in literature	- More than 1 h could be a useful cutoff based on results of 505 trial
			- May be reported as proportion of patients free from arrhythmia or time to recurrence
Freedom from AF/AFL/AT requiring intervention (emergency visits, cardioversion, urgent care visit, reablation, etc.)	- Can provide an endpoint more relevant to systemic costs of AF recurrence	- Will overestimate efficacy of ablation by ignoring shorter episodes not requiring intervention that still might be important to quality of life or stroke	- Determination of what is an “intervention” must be prespecified in protocol and biases mitigated to avoid over- or underintervention in the trial
	- Clinically relevant		
Freedom from persistent AF/AFL/AT-duration cutoff of 7 days	- Useful for trials assessing additional substrate modification in persistent AF	- Can systematically overestimate the efficacy of AF ablation, particularly for persistent AF	- Can require continuous monitoring to definitively assess if episode is >7 days
Freedom from AF/AFL/AT on previously ineffective antiarrhythmic therapy	- If patient maintains sinus rhythm on previously ineffective drug therapy, this may be considered a clinically relevant, successful outcome	- Will increase the success rate compared with off-drug success	- Postablation drug and dosage of drug should be identical to preablation drug and dosage
		- May not be relevant to patients hoping to discontinue drug therapy	
Significant reduction in AF burden: >75% reduction from pre- to postablation and/or total postablation burden <12%	- Can be useful in persistent AF studies, but might not be suited for early, paroxysmal AF studies	- Ideally requires continuous monitoring using an implantable device	- AF burden can be estimated by intermittent monitoring and reporting of patient symptoms and recurrences like a “time in therapeutic range” report for oral anticoagulation; see text
		- No scientific basic exists showing that a 75% reduction in AF burden impacts hard endpoints, including heart failure, stroke, and mortality	
			- Could also see 75% reduction in number and duration of AF episodes
			- Because there is no firm scientific basis for selecting the cutoff of 75%, this prior recommendation is provided only as an example of what future clinical trials may choose to use as a definition of clinical/partial success
Prevention in AF progression: time to first episode of persistent AF (>7 days)	- Does not assume that total elimination of AF is required	- Prevention in progression might be irrelevant for stroke or thromboembolic outcomes	- Might be useful for specific populations such as heart failure or hypertrophic cardiomyopathy, in which progression to persistent AF can lead to increased hospitalization
	- Well suited for paroxysmal or “early” AF studies in which goal is to prevent progression to persistent AF	- Long follow-up time might be required unless population is “enriched”	
		- Can ideally require continuous implantable monitoring	
Regression of AF: reduction in burden to a given threshold or conversion of persistent to paroxysmal AF	- Does not assume that total elimination of AF is required	- Regression endpoint will overestimate efficacy of AF ablation	- Could be particularly useful for long-standing persistent AF populations with structural heart disease, heart failure, etc.
	- Well suited for persistent “late” AF studies in which goal is to regress to paroxysmal AF, which might be easier to control with drug therapy	- Might ideally require continuous implantable monitoring	
		- Patients will require ongoing drug therapy	
Acute AF termination during ablation procedure	- Could provide indication of successful modification of substrate responsible for maintaining AF, most relevant to persistent or long-standing persistent AF	- Relevance of acute AF termination has not consistently been shown to correlate to long-term success	- Intraprocedural administration of preprocedural oral antiarrhythmics or intraprocedural intravenous antiarrhythmics are discouraged
	- Limited studies have linked acute AF termination to long-term success	- Endpoint might not be relevant to paroxysmal AF patients in whom AF might terminate spontaneously	- If antiarrhythmics are used, their use and dosage before and during the ablation should be clearly documented
	- Studies consider termination as reversion to sinus rhythm, whereas others consider reversion to any regular tachycardia as termination	- Some studies employ administration of intravenous or oral antiarrhythmics during ablation that could cause spontaneous termination	- Termination to sinus rhythm and termination to another regular tachycardia (AT or AFL) should be separately reported

*AF* atrial fibrillation, *AFL* atrial flutter, *AT* atrial tachycardia

### Unanswered questions in AF ablation

There is still much to be learned about the mechanisms of AF, techniques of AF ablation, and long-term outcomes. The following are unanswered questions for future investigation:

1. AF ablation and modification of stroke risk and need for ongoing oral anticoagulation (OAC): The CHA_2_DS_2_-VASc score was developed for patients with clinical AF. If a patient has received a successful ablation such that he/she no longer has clinical AF (subclinical, or no AF), then what is the need for ongoing OAC? Are there any patients in whom successful ablation could lead to discontinuation of OAC?
2. Substrate modification in catheter-based management of AF—particularly for persistent AF: What is the proper lesion set required beyond pulmonary vein isolation? Do lines and complex fractionated atrial electrogram (CFAE) have any remaining role? Are these approaches ill-advised or simply discouraged?
  What is the role of targeting localized rotational activations? How do we ablate a localized rotational activation? How can scar be characterized and targeted for ablation? Do we need to replicate the MAZE procedure? Does the right atrium need to be targeted as well as the left atrium?
3. Autonomic influence in AF: Is clinical AF really an autonomic mediated arrhythmia? Is elimination of ganglionated plexi required? Is there a role for autonomic modulation, for example, spinal cord or vagal stimulation?
4. Contribution and modulation of risk factors on outcomes of AF ablation: Obesity reduction has been shown to reduce AF burden and recurrence in patients undergoing ablation. What is the role of bariatric surgery? Does the modulation of other risk factors influence outcome such as hypertension, sleep apnea, and diabetes?
5. Outcomes in ablation of high-risk populations: Do high-risk populations benefit from AF ablation? Congestive heart failure has been assessed in smaller trials, but larger trials are required. Outcome data are needed in patients with very enlarged LAs, hypertrophic cardiomyopathy, patients with renal failure on dialysis, and the very elderly.
6. Surgical vs catheter-based vs hybrid ablation: There should be more comparative work between percutaneous and minimally invasive surgical approaches. Both report similar outcomes, but there is a dearth of comparative data. Is there any patient benefit to hybrid procedures?
7. How do we characterize patients who are optimal candidates for ablation? Preablation late gadolinium-enhanced (LGE)-magnetic resonance imaging (MRI) might identify patients with heavy burdens of scar who are unlikely to respond to ablation. These techniques must become reproducible and reliable and must be assessed in multicenter trials. Other markers need to be investigated, including genetic markers, biochemical markers, and clinical markers based on aggregated risk scores.
8. The incremental role of new technologies: As newer and often more expensive technologies are produced for AF ablation, their definitive incremental value must be determined in order to justify change in practice or case cost. These technologies include global (basket) mapping techniques, newer ablation indices for assessing lesion durability, advanced imaging for viewing lesions in the myocardium, etc. New energy sources, including laser, low-intensity ultrasound, photonic particle therapy, external beam ablation, and MRI-guided ablation, must be assessed in comparative fashion.
9. Outcomes of AF ablation: We need to better understand the clinical relevance of ablation outcomes. What is the significance of time to recurrence of 30 s of arrhythmia? How do we best quantify AF burden? How do these outcomes relate to quality of life and stroke risk?
10. What is the role of surgical LA reduction? Does left atrial appendage (LAA) occlusion or obliteration improve outcome of persistent AF ablation with an accompanying reduction in stroke? Does ablation work through atrial size reduction? What is the incidence of “stiff atrial” syndrome and does this mitigate the clinical impact of ablation?
11. Working in teams: What is the role of the entire heart team in AF ablation? Does a team approach achieve better outcomes than a “silo” approach?
12. Improving the safety of catheter ablation: As ablation extends to more operators and less experienced operators, the statistical occurrence of complications will increase. We need newer techniques to minimize complications and institute standards for operators to improve the reproducibility of ablation results and safety profiles at a variety of centers worldwide.
13. How does catheter ablation affect mortality, stroke, and hospitalization in broad and selected patient populations receiving catheter ablation for AF?
14. Management of patients who fail initial attempts at catheter ablation: Should there be specific criteria for repeat ablations (e.g., atrial size, body mass index)? Should patients be referred for surgery for repeat ablation?

In order to address these and other important questions in the field of catheter and surgical AF ablation, we urge investigators to create and participate in multisite collaborations and electrophysiology research networks with involvement of senior and junior investigators on the steering committees to push forward the next phase of AF research. We also urge funding bodies to support these important initiatives.

## Conclusion

Catheter ablation of AF is a very commonly performed procedure in hospitals throughout the world. This document provides an up-to-date review of the indications, techniques, and outcomes of catheter and surgical ablation of AF. Areas for which a consensus can be reached concerning AF ablation are identified, and a series of consensus definitions have been developed for use in future clinical trials of AF ablation. Also included within this document are recommendations concerning indications for AF ablation, technical performance of this procedure, and training. It is our hope to improve patient care by providing a foundation for those involved with care of patients with AF as well as those who perform AF ablation. It is recognized that this field continues to evolve rapidly and that this document will need to be updated. Successful AF ablation programs optimally should consist of a cooperative team of cardiologists, electrophysiologists, and surgeons to ensure appropriate indications, procedure selection, and follow-up.

## Appendix

Table 14

**Table 14:** Author disclosure table

Writing group member	Institution	Consultant/Advisory board/Honoraria	Speakers’ bureau	Research grant	Fellowship support	Stock options/Partner	Board Mbs/Other
Hugh Calkins, MD (Chair)	Johns Hopkins Medical Institutions, Baltimore, MD	1: Abbott Laboratories, 1: AtriCure, Inc., 1: Boston Scientific Corp., 1: Pfizer Inc., 1: St. Jude Medical, 1: Toray Industries Inc., 2: iRhythm, 3: Boehringer Ingelheim, 3: Medtronic, Inc.	None	2: Medtronic, Inc., 2: Boston Scientific Corp.	None	None	None
Gerhard Hindricks, MD (Vice-Chair)	Heart Center Leipzig, Leipzig, Germany	None	None	1: SIEMENS, 3: Biosense Webster, Inc., 3: Stereotaxis, Inc., 4: BIOTRONIK,5: Boston Scientific Corp., 5: St. Jude Medical	None	None	None
Riccardo Cappato, MD (Vice-Chair)	Humanitas Research Hospital, Arrhythmias and Electrophysiology Research Center, Milan, Italy*	None	None	None	None	None	None
Young-Hoon Kim, MD, PhD (Vice-Chair)	Korea University, Seoul, South Korea	None	1: St. Jude Medical	2: St. Jude Medical	None	None	None
Eduardo B. Saad, MD, PhD (Vice-Chair)	Hospital Pro-Cardiaco and Hospital Samaritano, Botafogo, Rio de Janeiro, Brazil	None	None	None	None	None	None
Luis Aguinaga, MD, PhD	Centro Privado de Cardiología, Tucuman, Argentina	None	None	None	None	None	None
Joseph G. Akar, MD, PhD	Yale University School of Medicine, New Haven, CT	1: Biosense Webster	None	None	None	None	None
Vinay Badhwar, MD	West Virginia University School of Medicine, Morgantown, WV	None	None	None	None	None	None
Josep Brugada, MD, PhD	Cardiovascular Institute, Hospital Clínic, University of Barcelona, Catalonia, Spain	None	None	None	None	None	None
John Camm, MD	St. George's University of London, London, United Kingdom	1: Actelion Pharmaceuticals, 1: Daiichi-Sankyo, 1: Eli Lilly, 1: Gilead Sciences, Inc., 1: Heart Metabolics, 1: InCarda Therapeutics, 1: InfoBionic, 1: Johnson and Johnson, 1: Medtronic, Inc., 1: Milestone, 1: Pfizer, Inc., 2: Boehringer Ingelheim, 2: Boston Scientific Corp., 2: Novartis 3: Bayer HealthCare, LLC	1: Daiichi-Sankyo, 1: Servier, 2: Bayer/Schering Pharma, 2: Boehringer Ingelheim	3: Boehringer Ingelheim, 3: Daiichi-Sankyo, 3: Pfizer, Inc.	None	None	0: European Heart Rhythm Association, 1: Oxford
Peng-Sheng Chen, MD	Indiana University School of Medicine, Indianapolis, IN	None	None	5: National Institutes of Health	None	5: Arrhythmotech	None
Shih-Ann Chen, MD	National Yang-Ming University, Taipei, Taiwan	1: Bayer/Schering Pharma, 1: Biosense Webster, 1: Boehringer Ingelheim, 1: Boston Scientific Corp., 1: Daiichi-Sankyo, 1: Medtronic Inc., 1: Pfizer Inc., 1: St. Jude Medical	1: St. Jude Medical	2: Biosense Webster, 2: St. Jude Medical	None	None	None
Mina K. Chung, MD	Cleveland Clinic, Cleveland, OH	0: Amarin, 0: BIOTRONIK, 0: Boston Scientific Corp., 0: Medtronic, Inc., 0: St. Jude Medical, 0: Zoll Medical Corporation	1: American College of Cardiology	None	None	None	1: Up to Date
Jens Cosedis Nielsen, DMSc, PhD	Aarhus University Hospital, Skejby, Denmark	None	None	5. Novo Nordisk Foundation	None	None	None
Anne B. Curtis, MD	University at Buffalo, Buffalo, NY	1: Daiichi-Sankyo, 1: Medtronic, Inc., 1: Projects in Knowledge, 2: St. Jude Medical	None	None	None	None	None
D. Wyn Davies, MD	Imperial College Healthcare NHS Trust, London, United Kingdom	1: Boston Scientific Corp., 1: Janssen Pharmaceuticals, 1: Medtronic, Inc., 1: Rhythmia Medical	None	None	None	3: Rhythmia Medical	None
John D. Day, MD	Intermountain Medical Center Heart Institute, Salt Lake City, UT	1: BIOTRONIK, 1: Boston Scientific Corp., 3: St. Jude Medical	None	None	None	None	None
André d’Avila, MD, PhD	Hospital SOS Cardio, Florianopolis, SC, Brazil	None	0: BIOTRONIK, 0: St. Jude Medical	0: BIOTRONIK, 0: St. Jude Medical	None	None	None
N.M.S. (Natasja) de Groot, MD, PhD	Erasmus Medical Center, Rotterdam, the Netherlands	None	None	None	None	None	None
Luigi Di Biase, MD, PhD	Albert Einstein College of Medicine, Montefiore-Einstein Center for Heart & Vascular Care, Bronx, NY	1: Atricure, 1: Biosense Webster, Inc., 1: BIOTRONIK, 1: Boston Scientific Corp., 1: EpiEP, 1: Medtronic, Inc., 1: St. Jude Medical, 1: Stereotaxis, Inc.	None	None	None	None	None
Mattias Duytschaever, MD, PhD	Universitair Ziekenhuis Gent (Ghent University Hospital), Ghent, Belgium	None	None	None	None	None	None
James R. Edgerton, MD	The Heart Hospital, Baylor Plano, Plano, TX	2: AtriCure, Inc.	1: AtriCure, Inc.	2: AtriCure, Inc.	None	None	None
Kenneth A. Ellenbogen, MD	Virginia Commonwealth University School of Medicine, Richmond, VA	1: American Heart Association, 1: Heart Rhythm Society, 2: Boston Scientific Corp.	1: AtriCure, Inc., 1: Biosense Webster, Inc., 1: BIOTRONIK, 1: St. Jude Medical, 2: Boston Scientific Corp., 2: Medtronic, Inc.	2: Biosense Webster, Inc., 2: Daiichi-Sankyo, 2: National Institutes of Health, 4: Boston Scientific Corp., 4: Medtronic, Inc.	None	None	1: Elsevier, 1: Wiley-Blackwell
Patrick T. Ellinor, MD, PhD	Massachusetts General Hospital, Boston, MA	1: Bayer HealthCare, LLC, 1: Quest Diagnostics	None	1: Leducq Foundation, 3: American Heart Association, 3: National Institutes of Health, 5: Bayer HealthCare, LLC	None	None	None
Sabine Ernst, MD, PhD	Royal Brompton and Harefield NHS Foundation Trust, National Heart and Lung Institute, Imperial College London, London, United Kingdom	2: Biosense Webster, Inc.	None	4: Spectrum Dynamics	None	None	None
Guilherme Fenelon, MD, PhD	Albert Einstein Jewish Hospital, Federal University of São Paulo, São Paulo, Brazil	1: Biosense Webster, Inc., 1: BIOTRONIK, 1: St. Jude Medical	None	None	None	None	None
Edward P. Gerstenfeld, MS, MD	University of California, San Francisco, San Francisco, CA	1: Boehringer Ingelheim, 1: Boston Scientific Corp., 1: Medtronic, Inc., 1: St. Jude Medical	None	4: Biosense Webster, Inc., 4: St. Jude Medical	2: Biosense Webster, Inc., 2: BIOTRONIK, 2: Boston Scientific Corp., 2: Medtronic, Inc.	1: Rhythm Diagnostic Systems Inc.	None
David E. Haines, MD	Beaumont Health System, Royal Oak, MI	1: Lake Region Medical, 1: Terumo Medical Corp	None	None	None	None	1: Biosense Webster, Inc., 1: Boston Scientific Corp., 1: Medtronic, Inc., 1: St. Jude Medical
Michel Haissaguerre, MD	Hôpital Cardiologique du Haut-Lévêque, Pessac, France	None	None	None	None	None	None
Robert H. Helm, MD	Boston University Medical Center, Boston, MA	None	None	None	None	None	1: Boston Scientific Corp.
Elaine Hylek, MD, MPH	Boston University School of Medicine, Boston, MA	1: Bayer, 1: Boehringer Ingelheim, 1: Bristol-Myers Squibb, 1: Daiichi-Sankyo, 1: Medtronic, 1: Portola, 1: Pfizer	None	2: Janssen Pharmaceuticals	None	None	None
Warren M. Jackman, MD	Heart Rhythm Institute, University of Oklahoma Health Sciences Center, Oklahoma City, OK	1: ACT, 1: VytronUS, Inc., 2: Biosense Webster, Inc., 2: Boston Scientific Corp., 2: Spectrum Dynamics	1: BIOTRONIK, 1: St. Jude Medical, 2: Biosense Webster, Inc., 2: Boston Scientific Corp.	None	None	None	None
Jose Jalife, MD	University of Michigan, Ann Arbor, MI, the National Center for Cardiovascular Research Carlos III (CNIC) and CIBERCV, Madrid, Spain	1: Topera Medical	None	1: Medtronic, Inc.	None	None	None
Jonathan M. Kalman, MBBS, PhD	Royal Melbourne Hospital and University of Melbourne, Melbourne, Australia	None	1: Boston Scientific Corp., 1: Medtronic, Inc.	4: Medtronic, Inc.	3: St. Jude Medical, 4: Biosense Webster, Inc., 4: Medtronic, Inc.	None	2: Biosense Webster, Inc., 4: Boston Scientific Corp.
Josef Kautzner, MD, PhD	Institute for Clinical and Experimental Medicine, Prague, Czech Republic	1: Bayer/Schering Pharma, 1: Boehringer Ingelheim, 1: Boston Scientific Corp., 1: Daiichi-Sankyo, 1: Sorin Group, 1: St. Jude Medical, 1: Biosense Webster, Inc., 2: Medtronic, Inc.	1: BIOTRONIK1: Medtronic, Inc.1: St. Jude Medical	None	None	None	None
Hans Kottkamp, MD	Hirslanden Hospital, Dept. of Electrophysiology, Zurich, Switzerland	1: Biosense Webster, Inc., 1: Kardium	None	None	None	1: Kardium	None
Karl Heinz Kuck, MD, PhD	Asklepios Klinik St. Georg, Hamburg, Germany	1: Biosense Webster, Inc., 1: BIOTRONIK, 1: St. Jude Medical, 1: Stereotaxis, Inc.	None	1: Biosense Webster, Inc., 1: BIOTRONIK, 1: St. Jude Medical, 1: Stereotaxis, Inc.	None	1: Endosense	None
Koichiro Kumagai, MD, PhD	Heart Rhythm Center, Fukuoka Sanno Hospital, Fukuoka, Japan	None	None	None	None	None	None
Richard Lee, MD, MBA	Saint Louis University Medical School, St. Louis, MO	None	None	None	None	None	None
Thorsten Lewalter, MD, PhD	Dept. of Cardiology and Intensive Care, Hospital Munich-Thalkirchen, Munich, Germany	1: BIOTRONIK, 1: Medtronic, Inc., 1: St. Jude Medical	1: Abbott Vascular, 1: BIOTRONIK, 1: Medtronic, Inc., 1: St. Jude Medical	None	None	None	None
Bruce D. Lindsay, MD	Cleveland Clinic, Cleveland, OH	0: Medtronic, Inc., 1: Abbott Vascular, 1: Biosense Webster, Inc.	None	None	3: Boston Scientific Corp., 3: Medtronic, Inc., 3: St. Jude Medical	None	None
Laurent Macle, MD	Montreal Heart Institute, Department of Medicine, Université de Montréal, Montréal, Canada	1: Bayer HealthCare, LLC, 1: Biosense Webster, Inc., 1: Boehringer Ingelheim, 1: Bristol-Myers Squibb, 1: Medtronic, Inc., 1: Pfizer, Inc., 1: Servier, 1: St. Jude Medical	None	4: Biosense Webster, Inc., 5: St. Jude Medical	None	None	None
Moussa Mansour, MD	Massachusetts General Hospital, Boston, MA	1: Biosense Webster, Inc., 1: St. Jude Medical	None	4: Biosense Webster, Inc., 4: St. Jude Medical,5: Pfizer,5: Boehringer Ingelheim	None	4: NewPace Ltd.	None
Francis E. Marchlinski, MD	Hospital of the University of Pennsylvania, University of Pennsylvania School of Medicine, Philadelphia, PA	1: Abbot Medical; 1: Biosense Webster, Inc., 2: BIOTRONIK, 1: Medtronic, Inc., 1: Boston Scientific Corp., 1: St. Jude Medical	None	3: Medtronic, Inc., 4: Biosense Webster, Inc.	1: BIOTRONIK, 3: Boston Scientific Corp., 3: Medtronic, Inc., 4: Biosense Webster, Inc., 5: St. Jude Medical	None	None
Gregory F. Michaud, MD	Brigham and Women's Hospital, Boston, MA	1: Biosense Webster, Inc., 1: Boston Scientific Corp., 1: Medtronic, Inc., 1: St. Jude Medical	None	4: Biosense Webster, Inc., 4: Boston Scientific Corp.	None	None	None
Hiroshi Nakagawa, MD, PhD	Heart Rhythm Institute, University of Oklahoma Health Sciences Center, Oklahoma City, OK	2: Biosense Webster, Inc 1: Boston Scientific Corp., 2: Stereotaxis, Inc., 3: Japan Lifeline, 3: Fukuda Denshi	1: Medtronic, Inc, 2: Boston Scientific Corp., 1: Spectrum Dynamics	4: Biosense Webster, Inc.,2: Japan Lifeline,2: Affera	None	None	None
Andrea Natale, MD	Texas Cardiac Arrhythmia Institute, St. David's Medical Center, Austin, TX	1: Boston Scientific Corp., 1: Janssen Pharmaceuticals, 1: Medtronic, Inc., 1: St. Jude Medical, 2: Biosense Webster, Inc.	None	None	None	None	None
Stanley Nattel, MD	Montreal Heart Institute and Université de Montréal, Montreal, Canada, McGill University, Montreal, Canada, and University Duisburg-Essen, Essen, Germany	1: Merck Pharmaceuticals, 1: Xention Discovery	None	3: OMEICOS Therapeutics	None	None	0: Montreal Heart Institute/Inventor Patents
Ken Okumura, MD, PhD	Division of Cardiology, Saiseikai Kumamoto Hospital, Kumamoto, Japan	1: Biosense Webster, Inc., 1: Boehringer Ingelheim, 1: Bristol-Myers Squibb, 1: Medtronic, Inc., 2: Bayer/Schering Pharma, 3: Daiichi-Sankyo	None	2: Biosense Webster, Inc., 2: Medtronic, Inc.	None	None	None
Douglas Packer, MD	Mayo Clinic, Rochester, MN	0: Abbott Laboratories, 0: Abiomed, 0: Aperture Diagnostics, 0: Biosense Webster, Inc., 0: Boston Scientific Corp., 0: CardioFocus, Inc., 0: CardioInsight Technologies, 0: Johnson and Johnson, 0: Johnson and Johnson Healthcare Systems, 0: MediaSphere Medical, LLC, 0: Medtronic CryoCath, 0: SIEMENS, 0: St. Jude Medical	None	0: American Heart Association, 0: Boston Scientific/EPT, 0: CardioInsight, 0: Endosense, 0: SIEMENS Acuson, 0: SIEMENS Acunav, 1: CardioFocus, 1: Hansen Medical, 1: Medtronic, Inc. 2: National Institutes of Health, 3: Thermedical (EP Limited), 5: Biosense Webster, 5: St. Jude Medical	None	None	1: Medtronic, 1: Oxford Press (Royalty), 1: SIEMENS, 1: WebMD, 1: Wiley-Blackwell (Royalty), 2: Biosense Webster, 4: St. Jude Medical (Royalty)
Evgeny Pokushalov, MD, PhD	State Research Institute of Circulation Pathology, Novosibirsk, Russia	1: Biosense Webster, Inc., 1: Boston Scientific Corp., 1: Medtronic, Inc.	None	None	None	None	None
Matthew R. Reynolds, MD, MSc	Lahey Hospital and Medical Center, Burlington, MA	1: Biosense Webster, Inc., 1: Medtronic, Inc., 1: St. Jude Medical	None	None	None	None	None
Prashanthan Sanders, MBBS, PhD	Centre for Heart Rhythm Disorders, South Australian Health and Medical Research Institute, University of Adelaide and Royal Adelaide Hospital, Adelaide, Australia	1: Biosense Webster, Inc., 1: Boston Scientific Corp., 1: CathRx, 1: Medtronic, Inc., 1: St. Jude Medical	1: Biosense Webster, Inc., 1: Boston Scientific Corp., 1: Medtronic, Inc., 1: St. Jude Medical	4: Sorin Group, 5: BIOTRONIK, 5: Boston Scientific Corp., 5: Medtronic, Inc., 5: St. Jude Medical	None	None	None
Mauricio Scanavacca, MD, PhD	Instituto do Coração (InCor), São Paulo, Brazil	1: Biosense Webster, Inc., 1: St. Jude Medical	1: Bayer/Schering Pharma, 1: Bristol-Myers Squibb, 1: Johnson and Johnson, 1: Daiichi-Sankyo	2: Johnson and Johnson	2: Johnson and Johnson	None	None
Richard Schilling, MD	Barts Heart Centre, London, United Kingdom	1: Biosense Webster, Inc., 1: Boehringer Ingelheim, 1: Daiichi-Sankyo, 1: Hansen Medical, 1: Medtronic, Inc., 1: St. Jude Medical	None	1: Boston Scientific Corp., 1: Hansen Medical, 1: Medtronic, Inc., 1: St. Jude Medical, 4: Boston Scientific Corp., 4: Medtronic, Inc., 4: St. Jude Medical	None	None	None
Claudio Tondo, MD, PhD	Cardiac Arrhythmia Research Center, Centro Cardiologico Monzino, IRCCS, Department of Cardiovascular Sciences, University of Milan, Milan, Italy	None	None	None	None	None	None
Hsuan-Ming Tsao, MD	National Yang-Ming University Hospital, Yilan City, Taiwan	None	None	None	None	None	None
Atul Verma, MD	Southlake Regional Health Centre, University of Toronto, Toronto, Canada	1: Bayer HealthCare, LLC, 1: Boehringer Ingelheim	None	5: Bayer HealthCare, LLC, 5: Biosense Webster, Inc., 5: BIOTRONIK, 5: Medtronic, Inc.	None	None	None
David J. Wilber, MD	Loyola University of Chicago, Chicago, IL	1: Biosense Webster, Inc., 1: Janssen Pharmaceuticals, 1: Medtronic, Inc., 1: St. Jude Medical, 1: Thermedical	None	1: Abbott Vascular, 1: Medtronic, Inc., 1: St. Jude Medical, 1: Thermedical, 3: Biosense Webster, Inc.	3: Biosense Webster, Inc., 3: Medtronic, Inc., 3: St. Jude Medical	None	1: Elsevier, 1: Wiley-Blackwell, 4: American College of Cardiology Foundation
Teiichi Yamane, MD, PhD	Jikei University School of Medicine, Tokyo, Japan	1: Bayer HealthCare, 1: Medtronic, 2: Abott Japan, 2: Daiichi-Sankyo, 2: Boehringer Ingelheim, 2: Bristol-Myers Squibb	None	1: Boehringer Ingelheim, 1: Bayer HealthCare	None	None	None

Number Value: 0 = $0; 1 = ≤ $10,000; 2 = > $10,000 to ≤ $25,000; 3 = > $25,000 to ≤ $50,000; 4 = > $50,000 to ≤ $100,000; 5 = > $100,000

*Dr. Cappato is now with the Department of Biomedical Sciences, Humanitas University, Milan, Italy, and IRCCS, Humanitas Clinical and Research Center, Milan, Italy

Table 15

**Table 15:** Reviewer disclosure table

Peer reviewer	Institution	Consultant/Advisory board/Honoraria	Speakers’ bureau	Research grant	Fellowship support	Stock options/Partner	Board Mbs/Other
Carina Blomström-Lundqvist, MD, PhD	Department of Cardiology and Medical Science, Uppsala University, Uppsala, Sweden	1: Bayer/Schering Pharma, 1: Boston Scientific Corp., 1: Medtronic, Inc., 1: Sanofi, 1: Pfizer, MSD, Bristol-Myers Squibb, Biosense Webster, Inc.	None	1: Cardiome Pharma/Astellas, 1: Medtronic, Inc.	None	None	None
Angelo A.V. De Paola, MD, PhD	Hospital São Paulo – Federal University of São Paulo, São Paulo, Brazil	None	None	None	None	None	None
Peter M. Kistler, MBBS, PhD	The Alfred Hospital Heart Centre, Melbourne, Australia	None	1: St. Jude Medical	None	None	None	None
Gregory Y.H. Lip, MD	University of Birmingham, Birmingham, United Kingdom; Aalborg University, Aalborg, Denmark	1: Medtronic,3: Bayer/Janssen, BMS/Pfizer, Boehringer Ingelheim, Daiichi-Sankyo	3: Bayer, BMS/Pfizer, Boehringer Ingelheim, Daiichi-Sankyo. No fees are received personally	None	None	None	None
Nicholas S. Peters, MD	St Mary's Hospital, Imperial College London, London, United Kingdom	1: Boston Scientific Corp., 1: Cardialen, Inc., 1: Cardiologs, 1: Magnetecs, 1: Medtronic, Inc., 1: St. Jude Medical	None	None	None	None	None
Cristiano F. Pisani, MD	InCor, Heart Insitute, HCFMUSP, Arrhythmia Unit	None	None	None	None	None	None
Antonio Raviele, MD	ALFA-Alliance to Fight Atrial Fibrillation, Rimini, Italy	None	None	None	None	None	None
Eduardo B. Saad, MD, PhD	Hospital Pro-Cardiaco and Hospital Samaritano, Botafogo, Rio de Janeiro, Brazil	None	None	None	None	None	None
Kazuhiro Satomi, MD, PhD	Tokyo Medical University, Tokyo, Japan	1: Bayer/Schering Pharma, 1: Boehringer Ingelheim, 1: Bristol-Myers Squibb, 1: Japan Lifeline, 1: Johnson and Johnson, 1: Medtronic, Inc., 1: Sankyo Pharmaceuticals, 1: St. Jude Medical	None	None	None	None	None
Martin K. Stiles, MB ChB, PhD	Waikato Hospital, Hamilton, New Zealand	1: Boston Scientific Corp., 1: Biosense Webster, Inc., 1: BIOTRONIK, 1: Medtronic, Inc.	None	None	1: Medtronic, Inc.	None	None
Stephan Willems, MD, PhD	University Medical Center Hamburg-Eppendorf, Hamburg, Germany	1: Bayer HealthCare, LLC, 1: Biosense Webster, Inc., 1: Boehringer Ingelheim, 1: Bristol-Myers Squibb, 1: Sanofi, 1: St. Jude Medical, 1: Medtronic	None	None	None	None	None

Number Value: 0 = $0; 1 = ≤ $10,000; 2 = > $10,000 to ≤ $25,000; 3 = > $25,000 to ≤ $50,000; 4 = > $50,000 to ≤ $100,000; 5 = > $100,000

## Abbreviations

AAD: Antiarrhythmic drug
AF: Atrial fibrillation
AFL: Atrial flutter
CB: Cryoballoon
CFAE: Complex fractionated atrial electrogram
LA: Left atrial
LAA: Left atrial appendage
LGE: Late gadolinium-enhanced
LOE: Level of evidence
MRI: Magnetic resonance imaging
OAC: Oral anticoagulation
RF: Radiofrequency
